# Fecal microbiota transplantation alleviates cognitive impairment by improving gut microbiome composition and barrier function in male rats of traumatic brain injury following gas explosion

**DOI:** 10.3389/fmicb.2024.1485936

**Published:** 2024-11-01

**Authors:** Xinwen Dong, Yaguang Su, Zheng Luo, Cuiying Li, Jie Gao, Xiaofeng Han, Sanqiao Yao, Weidong Wu, Linqiang Tian, Yichun Bai, Guizhi Wang, Wenjie Ren

**Affiliations:** ^1^Department of Environmental and Occupational Health, School of Public Health, Xinxiang Medical University, Xinxiang, China; ^2^Institute of Trauma and Orthopedics, Xinxiang Medical University, Xinxiang, China; ^3^Department of Pathology, The Fifth Affiliated Hospital of Zhengzhou University, Zhengzhou, China; ^4^Institute of Health Central Plains, Xinxiang Medical University, Xinxiang, China

**Keywords:** gas explosion, cognitive impairment, microbiota-gut-brain axis, fecal microbiota transplantation, gut barrier function, 16S rRNA gene sequencing, metabolomics

## Abstract

**Background:**

Dysbiosis of gut microbiota (GM) is intricately linked with cognitive impairment and the incidence of traumatic brain injury (TBI) in both animal models and human subjects. However, there is limited understanding of the impact and mechanisms of fecal microbiota transplantation (FMT) on brain and gut barrier function in the treatment of TBI induced by gas explosion (GE).

**Methods:**

We have employed FMT technology to establish models of gut microbiota dysbiosis in male rats, and subsequently conducted non-targeted metabolomics and microbiota diversity analysis to explore the bacteria with potential functional roles.

**Results:**

Hematoxylin–eosin and transmission electron microscopy revealed that GE induced significant pathological damage and inflammation responses, as well as varying degrees of mitochondrial impairment in neuronal cells in the brains of rats, which was associated with cognitive decline. Furthermore, GE markedly elevated the levels of regulatory T cell (Tregs)-related factors interleukin-10, programmed death 1, and fork head box protein P3 in the brains of rats. Similar changes in these indicators were also observed in the colon; however, these alterations were reversed upon transfer of normal flora into the GE-exposed rats. Combined microbiome and metabolome analysis indicated up-regulation of *Clostridium_T* and *Allobaculum*, along with activation of fatty acid biosynthesis after FMT. Correlation network analysis indirectly suggested a causal relationship between FMT and alleviation of GE-induced TBI. FMT improved intestinal structure and up-regulated expression of tight junction proteins Claudin-1, Occludin, and ZO-1, potentially contributing to its protective effects on both brain and gut.

**Conclusion:**

Transplantation of gut microbiota from healthy rats significantly enhanced cognitive function in male rats with traumatic brain injury caused by a gas explosion, through the modulation of gut microbiome composition and the improvement of both gut and brain barrier integrity via the gut-brain axis. These findings may offer a scientific foundation for potential clinical interventions targeting gas explosion-induced TBI using FMT.

## 1 Introduction

As a critical public health concern in the coal mining industry, gas explosions (GE) pose a significant threat to the safety of workers in male mines and roadways (Shieh et al., 2019). Due to the complex nature of roadways, GE occurrences often result in various injuries to miners. Although there is evidence indicating a decrease in mortality rates among coal miners with advancements in mining technology, the disability rate remains high and the prognosis is poor (Li et al., 2021; Dickstein et al., 2020). The brain tissue serves as a crucial target organ for GE patients, and the shock wave generated during an explosion can lead to damage of brain tissue and subsequent traumatic brain injury (TBI) (Dong et al., 2022). Furthermore, GE-induced TBI presents diverse complexities; our previous studies have established an association between GE and TBI (Dong et al., 2020), while current research primarily focuses on lung injuries (Dong et al., 2021b; Zhang M. et al., 2022). Among these findings, it has been well-documented that GE is linked with neurobehavioral abnormalities and its impact on injuries is more pronounced in developing brains. This is due to markers of GE-induced damage easily crossing through the blood–brain barrier or other means during post-injury recovery periods, exerting detrimental effects on the brain (Dong et al., 2021a). The accumulating evidence from epidemiological and experimental studies has confirmed the impact of TBI on neuronal damage (Thapak et al., 2024), blood–brain barrier disruption (Wang et al., 2021), and other related effects. TBI can be categorized into primary injury and secondary injury (Barrett et al., 2021). Secondary brain injury refers to additional damage, such as pathological inflammation following the initial injury (Khellaf et al., 2019), often leading to neurodegenerative diseases and cognitive impairment (Willis et al., 2020). Furthermore, GE-induced TBI can result in cognitive dysfunction in patients with a poor prognosis, emphasizing the critical need to identify potential therapeutic targets for addressing cognitive impairment.

The neuronal inflammation and neurodegenerative conditions resulting from TBI have implications on gut function via bidirectional communication between the gut and brain pathways (Jameson et al., 2020). Furthermore, various metabolites produced by the gut microbiota impact brain tissue through multiple pathways (Cryan et al., 2019). This underscores the importance of maintaining homeostasis in the gastrointestinal tract as a potential therapeutic target for TBI (Hanscom et al., 2021; Lei et al., 2023). Currently, there is increasing recognition of employing diverse strategies to modulate beneficial populations within the gut microbiome, such as fecal microbiota transplantation (FMT), as a clinical intervention for restoring intestinal flora (Antushevich, 2020; Zhao et al., 2023). FMT serves as an effective method for addressing imbalances in intestinal flora and rebuilding the microecosystem within the intestines (Ooijevaar et al., 2019; Yuan et al., 2024). Additionally, FMT entails transferring donor-derived microbiota to recipients with aims to reshape symbiotic microbial communities in order to confer protective effects against TBI (Koszewicz et al., 2021). Research indicates that alterations in intestinal flora composition disrupts homeostasis within the “gut-brain axis,” exacerbating secondary injury following nervous system trauma (Yuan et al., 2021). Our recent findings reveal cognitive deficits and gut microbiota dysbiosis in rats exposed to GE (Dong et al., 2024), underscoring a need for further investigation into how FMT specifically treats GE-induced TBI. Additionally, gut bacteria and their metabolites are capable of alternatively activated macrophages (M2) (Capizzi et al., 2020), reducing neural inflammation (Mossad and Erny, 2020), and increasing macrophage activity to enhance immune response while ameliorating neural degeneration (Soriano et al., 2022). Recent studies on both human populations and laboratory models highlight close associations between FMT efficacy with respect to gut barrier function, microbiota composition, as well as bacterial metabolic product levels (Prochazkova et al., 2019; Ma et al., 2021). Nonetheless, limited knowledge exists regarding long-term effects of FMT treatment during recovery from GE-induced TBI.

The precise mechanisms underlying the connection between gut microbiota and the effects of brain injury have not yet been fully elucidated. Currently, it has been suggested that a stable composition of gut microbiota positively regulates long-term neurodegenerative processes following traumatic brain injury through the microbiota-gut-brain axis (Chiu and Anderton, 2023). Variations in gut microorganisms can be detected by toll-like receptors (TLRs), with TLR4 being particularly adept at recognizing bacterial endotoxins such as lipopolysaccharide (LPS) and triggering the production of proinflammatory cytokines, as demonstrated in my previous study (Dong et al., 2024). Subsequently, LPS and proinflammatory cytokines further disrupt intestinal barrier function, leading to an increase in permeability and translocation into systemic circulation, causing systemic inflammation. Ultimately, these substances may even breach the blood–brain barrier (BBB), affect regulatory T cells (Tregs)-related factors, and result in neuronal death (Paudel et al., 2020). In a stress-induced rat model of depressive-like behavior, the findings demonstrated that FMT treatment ameliorates depressive-like behavior, modulates gut microbiota imbalance, and mitigates intestinal tract inflammation, disruption of intestinal mucosa, and neuroinflammation in rats (Rao et al., 2021). “Further investigations are warranted to assess the potential role of the microbiota-gut-brain axis in mediating the efficacy of FMT treatment for GE-induced TBI, given the observed dysbiosis in gut microbiota following GE.

In this study, we investigated the neuroprotective effects of FMT on neurological deficits, neuropathological changes, and neuroinflammation in GE-induced TBI rats by modulating the microbiota-gut-brain axis. Additionally, we evaluated gut microbiota composition, gut and brain barrier function, as well as the population of Treg cells in the brain. Furthermore, we have confirmed that the potential anti-inflammatory mechanisms identified through integrated microbiome and metabolome profiling are involved in the peripheral immune pathway, providing a promising strategy to alleviate GE-induced TBI.

## 2 Materials and methods

### 2.1 Chemicals, reagents, and instruments

Neomycin sulfate (N109017), natamycin (P107822), and sodium butyrate (S102956) were procured from Shanghai Aladdin Biochemical Technology Co., Ltd. Bacitracin (S17005) was obtained from Shanghai Yuanye Bio-Technology Co., Ltd. RNA isolate Total RNA Extraction Reagent (R401-01) was purchased from Nanjing Vazyme Biotech Co., Ltd. The Protein Quantification Kit (BCA Assay) (KTD3001) and (PC0020) was acquired from Abbkine (Wuhan) Scientific Co., Ltd. and Beijing Solarbio Science and Technology Co., Ltd., respectively. BeyoECL Star (Ultra hypersensitive ECL chemiluminescence kit) (P0018AS) was sourced from Shanghai Beyotime Biotechnology Co., Ltd. Western blot and IHC revealed the presence of *β*-Actin (bs-0061R), Interleukin 10 (IL-10) (bs-0698R), programmed death 1 (PD-1) (bs-1867R), Fork head box protein P3 (Foxp3) (bs-10211R), Zonula occludens-1 (ZO-1) (bs-1329R), Occludin (bs-10011R). Claudin-1 (bs-1428 R) and Claudin-5 (bs-1241 R) were obtained from Biosynthesis Biotechnology lnc. (Beijing China).

### 2.2 Animals and experimental design

The study was conducted in compliance with international standards for the Feeding and use of laboratory animals. Forty healthy male Sprague–Dawley rats (100 ± 5) g were provided by Henan Skobes Biotechnology Co., Ltd, China (License key: SCXK(Yu)2020-0005). The SD rats were housed in standard cages and maintained under typical ambient conditions of temperature (21 ± 1°C) and relative humidity (50 ± 10%) on a regular 12 h light/12 h dark cycle. All rats underwent a one-week acclimatization period prior to formal experiments.

The rats were randomly divided into 5 groups (*n* = 8/group): the control group (CON) did not undergo any treatment; GE model group (MOD): exposed to GE using an Experimental System for Biological Lethality Testing of Gas Explosions in Shock Tubes designed by Huludao Northern Petrochemical Equipment Factory, China (Utility Model Patent Certificate: CN 214150529 U). Rats were anesthetized with pentobarbital and secured in an iron cage facing the explosion source. GE was simulated using a 10% methane mixture and a 23.6 J shock wave. The rats were positioned 2.4 meters away from the explosion source; GE + fecal microbiota transplantation group (FMT): following exposure to GE, the fecal microbiota suspension derived from normal rat feces was administered via gavage; Antibiotic clearance + GE group (ABX): before exposure to GE, the rats received combined antibiotics to eliminate intestinal flora, followed by normal feeding after exposure to GE; Antibiotic removal + GE + fecal microbiota transplantation group (AF): prior to exposure to GE, the rats received combined antibiotics for intestinal flora elimination, and after exposure to GE, the fecal microbiota suspension derived from normal rat feces was administered via gavage. A shock tube device was simulated a GE in the Institute of Trauma and Orthopedics, Xinxiang Medical University. After anesthesia, the rats were fixed in an explosion cage with their heads and faces facing the explosion source. Neomycin sulfate (5 mg/mL), natamycin (1.25 μg/mL), and bacitracin (5 mg/mL) were dissolved in rat drinking water to prepare combined antibiotic drinking water which was used for rats to achieve the purpose of antibiotic clearance refer to previous study (Bercik et al., 2011). Fresh feces of rats in the control group were collected and dissolved in normal saline to make fecal microbiota suspension. Feces were collected according to the volume to weight ratio of 1:5. This study was approved by the Animal and Medical Ethics Committee of Xinxiang Medical University (No. XYLL-2020007).

### 2.3 Sample collection and processing

The fecal samples from each rat were collected on the 21st day post-gavage and stored at −80°C for subsequent 16S rRNA gene sequencing. Subsequently, the body weight was recorded, and euthanasia was performed using 1% sodium pentobarbital. Blood was drawn from the abdominal aorta and incubated at 4°C for 2 h before centrifugation at 3000 rpm for 15 min. Following centrifugation, the serum was separated and stored in a refrigerator at −80°C. Brain and colon tissues were then harvested, with simultaneous removal of intestinal contents during colon tissue collection, which were also stored at −80°C for future use. In addition to macroscopic examination of the collected tissues, brain and colon tissues (*n* = 3) underwent hematoxylin eosin staining (H&E) as well as transmission electron microscopy (TEM). These procedures were conducted to facilitate subsequent observation of histopathological changes and assessment of tissue damage. The remaining tissues were preserved in a − 80°C freezer for molecular biology experiments.

### 2.4 Fecal microbiota transplantation

FMT was conducted following a well-established protocol (Chang et al., 2015). Briefly, fresh fecal samples were obtained from male rats in the Control group. The samples were collected using a sterile tube and weighed. Subsequently, 0.9% NaCl solution was added to the weighed feces four times, and the mixture was homogenized by stirring with a sterile cotton swab before being centrifuged at 1500 rpm for 5 min. The resulting pellet was then resuspended in 0.9% NaCl to achieve a microbial concentration of 2 × 10^9^ CFU/mL for FMT testing purposes. A fecal bacterial suspension of 1 mL/100 g was orally administered to each gastrointestinal model rat via gavage on a daily basis for 21 consecutive days.

### 2.5 Blood pressure and heart rate test

Blood pressure and heart rate were measured prior to euthanasia of the rats. All rats in the five groups (*n* = 8) were tested sequentially. The rats were positioned in the detection device, and tail-end detection was performed with five repetitions for each rat. Data collection was carried out using a Smart Non-Invasive Blood Pressure Monitor (Softron BP-2010 Series, China), as per the operational manual, and statistical analysis was conducted using GraphPad Prism 8.

### 2.6 Open filed test

The open field test (OFT) apparatus comprises a black square substrate measuring 80 × 80 and a black wall spanning 50 cm. The substrate is divided into 16 equally spaced squares, with the central area consisting of 4 squares and the remaining 12 squares designated as the periphery area. Each rat is gently placed in the center of the apparatus and allowed to explore freely for 3 min before being captured. Subsequently, all rats are observed in an open field box for 5 min using a video tracking system. Following each session, the open field box is cleaned with a solution of 10% alcohol to mitigate any potential odor influence on subsequent rats. Locomotor activity is evaluated based on distance covered across the entire area. The total moving distance and average moving speed of the rats are utilized to assess neurobehavioral changes. Data collected during these experiments are recorded and analyzed using an animal trajectory tracking system (Etho Vision XT16.0, Noldus).

### 2.7 Hematoxylin and eosin staining and transmission electron microscope analysis

After sample collection, histopathological examination was conducted to assess the TBI induced by GE. Brain and colon tissues from each group of rats (*n* = 3) were previously fixed in 4% paraformaldehyde, followed by embedding, sectioning, staining, and mounting. Subsequently, H&E-stained samples were examined using an upright optical microscope (Nikon Eclipse E100, Japan), and the resulting images were captured and analyzed with an imaging system (NIKON DS-U3, Japan).

For TEM analysis, the brain and colon tissues of rats in each group (*n* = 3) were fixed with 2.5% glutaraldehyde solution. Each sample was sectioned into 1 mm^3 cubes and immersed in fixative at 4°C for 4 h. Subsequently, the tissues underwent fixation in 1% osmium tetroxide at room temperature for 2 h, followed by dehydration using a gradient alcohol series. The specimens were then embedded in resin and polymerized by baking at 60°C for 48 h. Ultra-thin sections (60 nm) were obtained using an ultramicrotome. Finally, ultrastructural examination of tight junctions in both brain and colon tissues was conducted using a transmission electron microscope (HITACHI, HT7700, Japan).

### 2.8 IHC staining and western blot assay

For IHC staining, brain and colon tissue sections were incubated with Claudin-1, Occludin, ZO-1, PD-1, Foxp3, and IL-10 antibodies (dilution 1:500) overnight at 4°C. Subsequently, the sections were treated with secondary antibodies (dilution 1:500) for 2 h at room temperature. Following this, the tissue sections underwent incubation in 0.003% hydrogen peroxide in 0.01 M PBS and 0.05% DAB for 10 min in darkness to visualize the immune response; then they were counterstained with hematoxylin for 5 min. The localization and distribution of immunoreactive substances in brain and colon tissues were examined under a microscope. The results were quantified as the percentage of positive area.

For western blot analysis, brain and colon tissues were placed on the operating table, sectioned with a blade, and then immersed in RIPA solution. Following homogenization, EP tubes were utilized for centrifugation to extract the supernatant, and protein concentrations were determined using the BCA kit. Subsequently, proteins underwent separation via sodium dodecyl sulfate-polyacrylamide gel electrophoresis before being transferred onto PVDF membranes. The membranes were incubated overnight at 4°C with primary antibodies including *β*-Actin, Claudin-1, Occludin, ZO-1, PD-1, Foxp3 and IL-10 (1:2000). Afterward, corresponding secondary antibodies such as HRP-conjugated anti-rabbit antibody (1:50000) were applied for 1 h followed by washing with TBST and development using an ECL kit. Visualization of blots was achieved utilizing the Amersham ImageQua system while density analysis was conducted using ImageJ software. Results were expressed as the gray value of target protein relative to the internal reference protein.

### 2.9 RNA extraction and real-time quantitative PCR

The total RNA was extracted using TRIzol reagent (R401-01, Vazyme Biotech Co., Ltd., Nanjing, China), and the concentration of the extraction was determined using a nucleic acid detector. Subsequently, 2 μL of total RNA was mixed with reverse transcriptase to synthesize cDNA using a gradient PCR apparatus. After 10-fold dilution of the cDNA, polymerase chain reaction was performed. The reaction system comprised 0.4 μL forward primer, 0.4 μL reverse primer, 7.2 μL enzyme-free water, 2 μL cDNA and 10 μL SYBR qPCR Master Mix in a total volume of 20 μL. Following the reaction, the number of cycles for each gene was obtained using a multifunctional microplate reader. Each sample underwent triplicate testing to ensure accuracy in determining the relative expression of the target gene in the sample. Results: The relative expression was obtained using the 2^−△△Ct^ method combined with *β*-Actin as reference. Primer names and sequences for mRNA detection are provided in Supplementary Table S1.

### 2.10 Intestinal contents DNA extraction, 16S rRNA gene sequencing, and Bioinformatic analysis

A total of 5 groups (*n* = 5) comprising 25 qualified samples of intestinal contents were utilized for DNA extraction. The genomic DNA was extracted using the OMEGA Soil DNA Kit (M5635-02) (Omega Bio-Tek, Norcross, GA, United States). Subsequently, the purity and concentration of the DNA were assessed using a NanoDrop NC2000 spectrophotometer (Thermo Fisher Scientific, Waltham, MA, United States) and agarose gel electrophoresis, respectively. Following this, an appropriate quantity of sample DNAs was taken in a centrifuge tube and diluted to 1 ng/μL with sterile water prior to PCR amplification. The bacterial 16S rRNA genes V3–V4 region was amplified via PCR utilizing the forward primer 338F (5’-ACTCCTACGGGAGGCAGC-3′) and reverse primer 806R (5’-GGACTACHVGGGTWTCTAAT-3′). The resulting 16S rRNA sequencing data were analyzed as previously described (Ma et al., 2020). Post individual quantification steps, amplicons were pooled equitably before undergoing pair-end 2 × 250 bp sequencing on the Illlumina NovaSeq platform with NovaSeq 6,000 SP Reagent Kit (500 cycles) at Shanghai Personal Biotechnology Co., Ltd. (Shanghai, China).

The analysis of microbiome bioinformatics was carried out using QIIME2 2022.11 with minor adjustments in accordance with the official tutorials.^1^ Sequence data were primarily processed utilizing QIIME2 and R packages (v3.2.0). Alpha diversity indices at the amplicon sequence variants (ASV) level, such as Chao1 richness estimator, observed species, Shannon diversity index, and Simpson index, were computed from the ASV table in QIIME2 and presented as box plots. ASV-level ranked abundance curves were generated to compare the richness and evenness of ASVs across samples. Beta diversity analysis was conducted to explore the structural variation of microbial communities among samples using Bray-Curtis’s metrics, visualized through principal coordinate analysis (PCoA). Additionally, Principal component analysis (PCA) was performed based on genus-level compositional profiles. The differentiation of microbiota structure among groups was assessed for significance utilizing PERMANOVA (Permutational multivariate analysis of variance) within QIIME2. Furthermore, LEfSe (Linear discriminant analysis effect size) was utilized to identify differentially abundant taxa across groups with default parameters. Microbial functions were predicted by employing PICRUSt2 (Phylogenetic investigation of communities by reconstruction of unobserved states) based on MetaCyc^2^ and KEGG^3^ databases.

### 2.11 Non-targeted metabolomics analysis of the fecal

The fecal samples (*n* = 5/group) from rats in different treatment groups underwent non-targeted metabolomics analysis using ultra-high-performance liquid chromatography–tandem mass spectrometry (UPLC-MS/MS) at Personalbio Technology Co., Ltd., as previously outlined (Li et al., 2022). The processed data was assessed for overall differences among the three groups utilizing Pareto-scaled principal component analysis (PCA) and orthogonal partial least-squares discriminant analysis (OPLS-DA). Metabolites with a variable importance in projection (VIP) value >1 and a *p*-value <0.05 (two-tailed Student’s *t*-test) were deemed significant, indicating potentially relevant metabolic markers. The Kyoto Encyclopedia of Genes and Genomes (KEGG) database was employed to investigate related metabolic and signal transduction pathways linked to significantly differentially expressed metabolites in the various treatment groups. This study was carried out by Shanghai Personal Biotechnology Co., Ltd., Shanghai, China.

### 2.12 Statistical analysis

The mean ± standard deviation (SD) was used to express all data, and GraphPad Software (GraphPad Prism version 8, La Jolla, CA, United States) was employed to create statistical charts. Statistical analysis was performed using SPSS software (version 20.0). Group differences were assessed through one-way and two-way analysis of variance (ANOVA) followed by Turkey’s multiple comparisons tests. The independent *t* test was applied for comparing two groups. Spearman correlation analysis conducted by R (V3.5.1) was used to determine the correlations among different groups. In addition, species were clustered by default, that is, UPGMA clustering (default clustering algorithm) was performed according to the Pearson correlation coefficient matrix of their component data, and arranged according to the clustering results. Results with a *p* value <0.05 were considered statistically significant.

## 3 Results

### 3.1 FMT facilitates the amelioration of cognitive dysfunction and brain barrier impairment induced by GE

**Figure 1:**
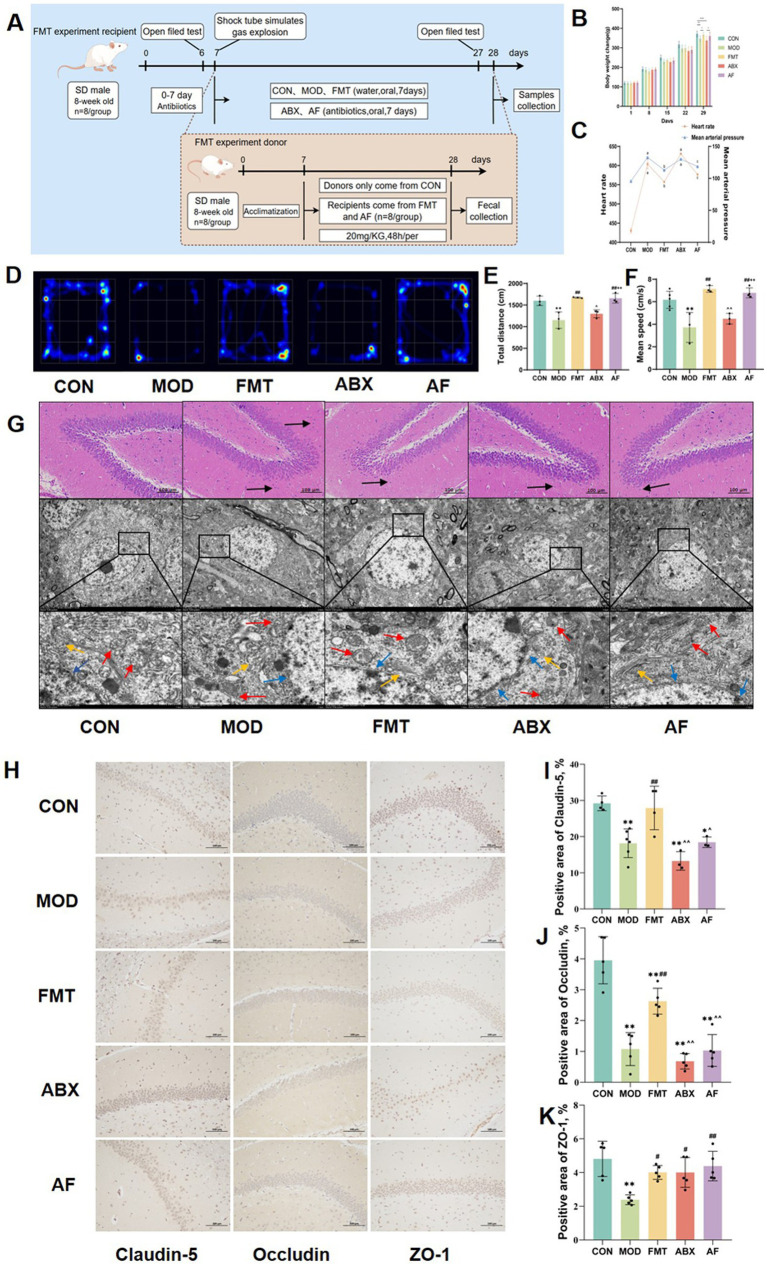
FMT facilitates the amelioration of cognitive impairment and blood–brain barrier injury induced by GE. The study included five groups: control group (CON), GE model group (MOD), GE+ fecal microbiota transplantation group (FMT), antibiotic clearance + GE group (ABX) and antibiotic removal + GE + fecal microbiota transplantation group (AF). **(A)** Flow chart depicting the fecal microbiota transfer experiment. **(B)** Body weight change observed in each group. **(C)** Heart rate and mean arterial pressure measured in each group. **(D)** Heatmap illustrating open field test results for each group. **(E,F)** Total distance traveled and mean speed of each group. **(G)** H&E staining and TEM scanning of dentate gyrus of the hippocampus conducted. **(H)** The tight junction proteins in striatum were detected by IHC (*n* = 3). **(I–K)** Positive area of junction proteins (Claudin-5, Occludin5, and ZO-1) in the striatum quantified using image analysis software. The data represent the mean ± SD, *p* < 0.05 was set as the threshold for significance by one-way and two-way ANOVA followed by *post hoc* comparisons using Tukey’s test for multiple groups’ comparisons. * Indicates statistical significance compared to the CON group, * *p* < 0.05, ** *p* < 0.01, # indicates statistical significance compared to the MOD group, # *p* < 0.05, ## *p* < 0.01, ^ indicates statistical significance compared to the FMT group, ^ *p* < 0.05, ^^ *p* < 0.01, +indicates statistical significance compared to the ABX group, ++ *p* < 0.01. Black arrows point to the inflammatory cells, blue arrows point to the nuclear membrane structure, red arrows point to the mitochondria, yellow arrows point to the endoplasmic reticulum. The histological sections are from the same anatomical regions, but at completely different coordinates. OFT experiment repeated 3 times, IHC experiment repeated 5 times.

To investigate the potential impact of intestinal microbiota on ameliorating brain injury in rats with traumatic brain injury (TBI) induced by gastric aspiration, we administered antibiotics to the ABX and AF groups, while fecal bacteria from rats in the CON group were transplanted into the FMT and AF groups (Figure 1A). Following gastric aspiration, abnormal body weight, heart rate, and blood pressure were observed in the rats compared to the CON group; however, these parameters improved following FMT (Figures 1B,C). The results of open field tests (Figures 1D–F) revealed reduced movement distance and speed in MOD and ABX groups compared to the CON group, whereas these impairments were mitigated after FMT in the FMT and AF groups. These findings suggest a potential role for gut microbiota in alleviating cognitive impairment associated with gastric aspiration.

To further investigate the cerebral injury induced by GE and assess potential improvements following FMT, we conducted an assessment of the dentate gyrus of the hippocampus using H&E staining and TEM scanning (Figure 1G). The H&E staining revealed a reduction in cell numbers with loose arrangement in the MOD and ABX groups. Following FMT, there was an increase in cell numbers with darker coloration observed in the FMT and AF groups. TEM scanning demonstrated reduced cell numbers, mitochondrial swelling, cavitation, as well as dilated and degranulated endoplasmic reticulum in the MOD and ABX groups. Post-FMT observations included intact nuclear membrane structure, reduced mitochondrial count with visible cristae, along with persistent dilation of endoplasmic reticulum in both FMT and AF groups.

To further assess striatal damage, we examined alterations in the expression of tight junction proteins within the striatum (Figures 1H–K). In comparison to the CON group, there was a significant reduction in the positive area ratio of Claudin-5 and Occludin protein (Figures 1I,J) in the MOD and ABX groups (*p* < 0.001). Following FMT, these ratios increased in the FMT group (*p* < 0.01), while no significant change was observed in the AF group (Figure 1K). Notably, there were no substantial differences in ZO-1 protein expression levels (Figure 1K). Our findings demonstrate that GE can induce blood–brain barrier damage, which is ameliorated by FMT treatment, thereby exerting a protective effect.

### 3.2 FMT induced the upregulation of regulatory T cell-related factors proteins expression in the striatum

**Figure 2:**
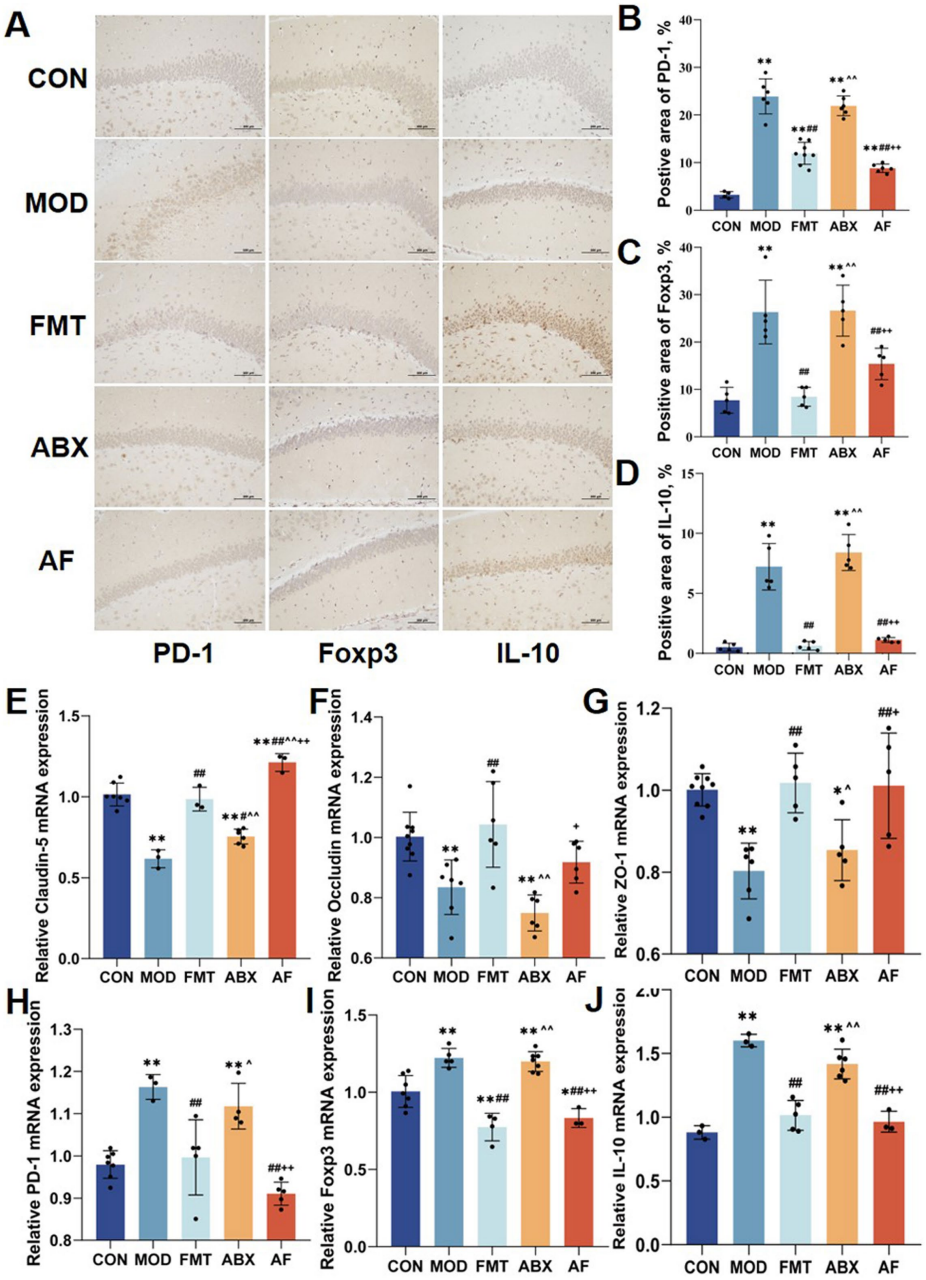
FMT prompted the striatum Tregs cell factor expression. There were five groups: control group (CON), GE model group (MOD), GE+ fecal microbiota transplantation group (FMT), antibiotic clearance + GE group (ABX) and antibiotic removal + GE + fecal microbiota transplantation group (AF). **(A)** Tregs associated factors proteins in striatum were detected by IHC (*n* = 3). **(B–D)** Positive area of Tregs-associated factors proteins (PD-1, Foxp3 and IL-10) in the striatum (*n* = 3). **(E–J)** Relative mRNA levels with *β*-actin of the junction proteins (Claudin-5, Occludin and ZO-1) and regs-associate factors (PD-1, Foxp3 and IL-10) in striatum (*n* = 6). The data represent the mean ± SD, *p* < 0.05 was set as the threshold for significance by one-way and two-way ANOVA followed by *post hoc* comparisons using Tukey’s test for multiple groups’ comparisons. * Indicates statistical significance compared to the CON group, * *p* < 0.05, ** *p* < 0.01, # indicates statistical significance compared to the MOD group, # *p* < 0.05, ## *p* < 0.01, ^ indicates statistical significance compared to the FMT group, ^ *p* < 0.05, ^ ^ *p* < 0.01, +indicates statistical significance compared to the ABX group, + *p* < 0.05, ++ *p* < 0.01. IHC experiment repeated 5 times, RT-PCR experiment repeated more than 3 times.

To investigate whether the protective effect of FMT against GE-induced blood–brain barrier damage is associated with immune system regulation *in vivo*, we assessed changes in the immunohistochemical expression of regulatory T cell (Tregs)-related proteins in the striatum (Figures 2A–D). Compared to the control group, there was a significant increase in the positive area ratio of PD-1, Foxp3, and IL-10 proteins (Figures 2B–D) in the model and antibiotic-treated groups; following FMT administration, these ratios decreased in the FMT and antibiotic-free groups (*p* < 0.001). Furthermore, to examine tight junction protein and Tregs-related factor expression at the mRNA level, we analyzed their transcript levels (Figures 2E–J). The expression patterns of these six indicators were largely consistent with those observed at the protein level.

### 3.3 FMT facilitated the amelioration of gastrointestinal barrier injury induced by GE

**Figure 3:**
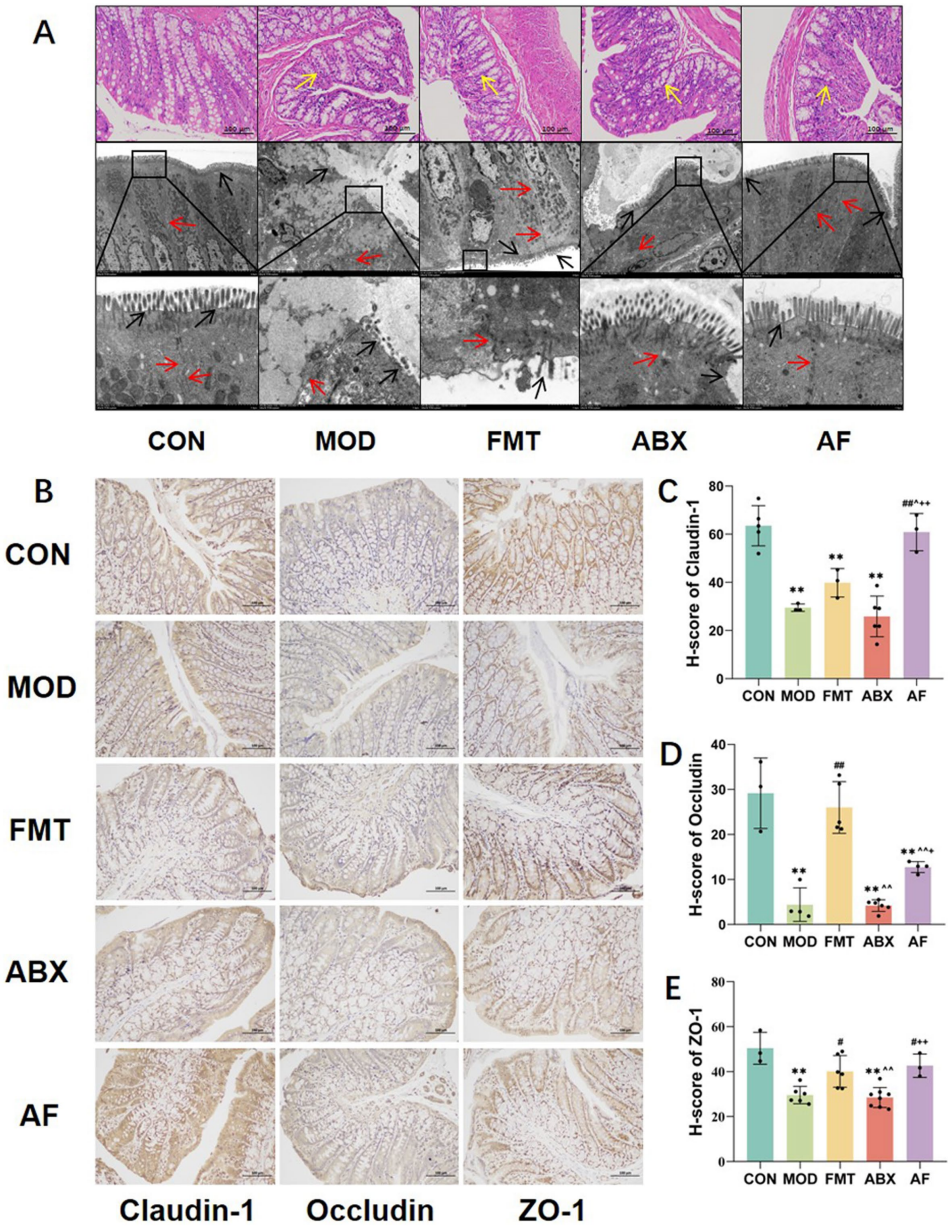
FMT facilitated the amelioration of intestinal barrier injury induced by GE. The study included five groups: control group (CON), GE model group (MOD), GE+ fecal microbiota transplantation group (FMT), antibiotic clearance +GE group (ABX), and antibiotic removal +GE+ fecal microbiota transplantation group (AF). **(A)** H&E staining and TEM scanning of the colon were performed. **(B)** Immunohistochemistry was used to detect the junction proteins in the colon (*n* = 3). **(C–E)** The H-score of the junction proteins, including Claudin-1, Occludin, and ZO-1, in the colon was calculated (*n* = 3). The data represent the mean ± SD, *p* < 0.05 was set as the threshold for significance by one-way and two-way ANOVA followed by *post hoc* comparisons using Tukey’s test for multiple groups’ comparisons. * Indicates statistical significance compared to the CON group, ** *p* < 0.01, # indicates statistical significance compared to the MOD group, # *p* < 0.05, ## *p* < 0.01, ^ indicates statistical significance compared to the FMT group, ^ *p* < 0.05, ^^ *p* < 0.01, +indicates statistical significance compared to the ABX group, + *p* < 0.05, ++ *p* < 0.01. Yellow arrows point to intestinal glands, black arrows point to intestinal villous structures and red arrows point to cell junctions. The histological sections are from the same anatomical regions, but at completely different coordinates. IHC experiment repeated 5 times.

To assess the impact of GE on gut health and to investigate the direct influence of gut microbiota, we conducted histological evaluation of colon injury using H&E staining and TEM scanning (Figure 3A). In the MOD and ABX groups, decreased number of intestinal epithelial cells, intestinal glands exhibited irregular curvature and disorganized arrangement, and there was incomplete connective tissue. Following FMT treatment, cellular structure and numbers in the FMT group showed recovery; however, restoration of intestinal glands and connective tissues was not pronounced. In the AF group, cellular structure and numbers improved but upper mucosal damage persisted with limited recovery of intestinal glands and connective tissues. TEM scanning revealed desquamation of intestinal villi, damaged boundaries, and severe disruption of cell tight junctions in the MOD and ABX groups. Post-FMT treatment, intestinal villi in both FMT and AF groups displayed regrowth with relatively intact intercellular connections; nevertheless, these connections remained somewhat blurred.

To further confirm intestinal injury, we evaluated alterations in the expression of tight junction proteins in intestinal cells (Figures 3B–E). Our findings revealed that compared with the CON group, the H-scores of Claudin-5, Occludin, and ZO-1 proteins (Figures 3C–E) were significantly reduced in the MOD and ABX groups (*p* < 0.001). Following FMT, the expression levels in the FMT and AF groups exhibited varying degrees of improvement. Our experiments have demonstrated that gastrointestinal endotoxemia can induce damage to the intestinal barrier, while fecal microbiota transplantation directly ameliorates this impairment.

### 3.4 FMT ameliorated GE-induced intestinal inflammation and enhanced the expression of regulatory T cell factors in the colon

**Figure 4:**
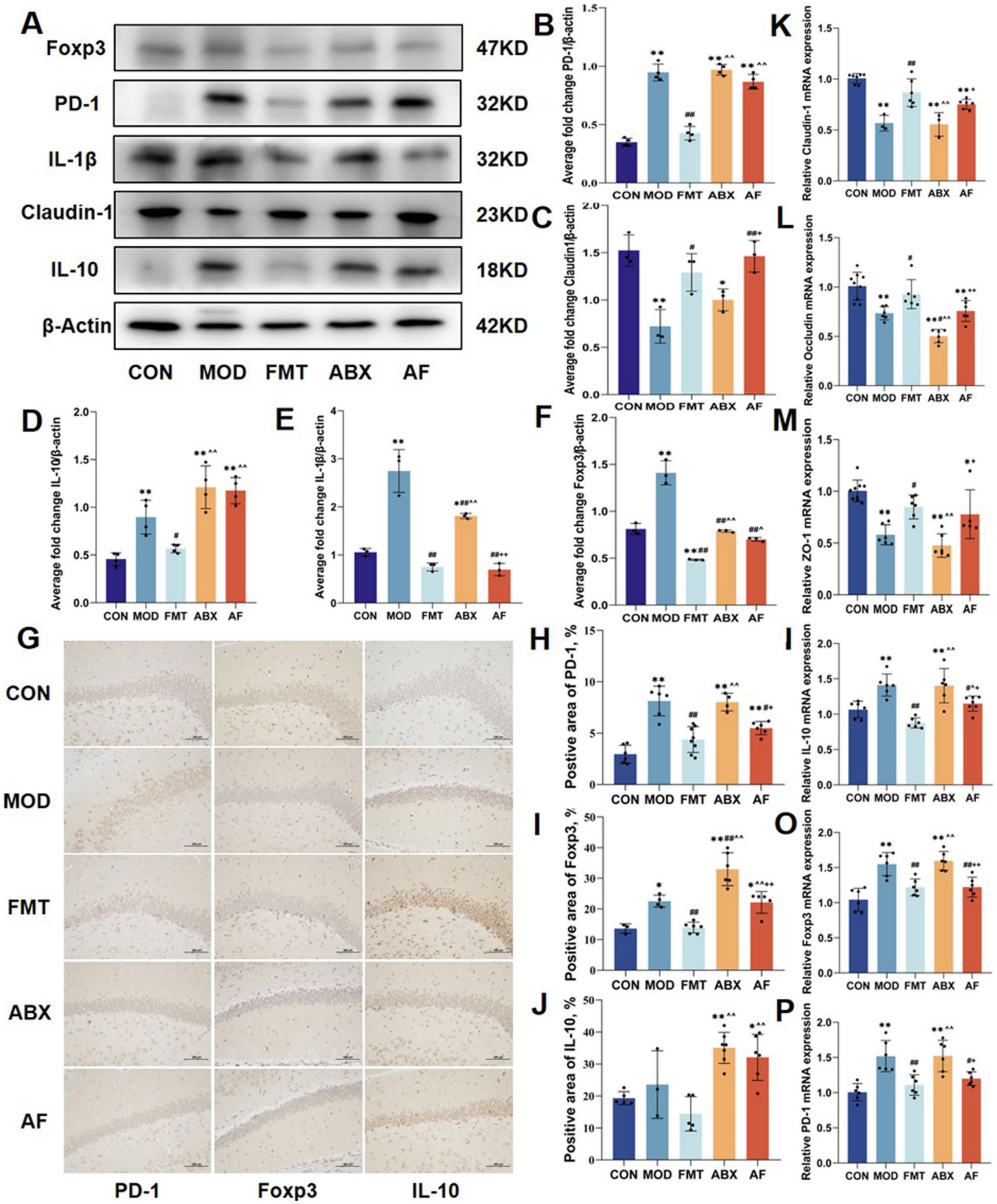
FMT enhanced GE induced by intestinal inflammation and stimulated the expression of colon Tregs cell factors. The study included five groups: control group (CON), GE model group (MOD), GE + fecal microbiota transplantation group (FMT), antibiotic clearance +GE group (ABX), and antibiotic removal +GE+ fecal microbiota transplantation group (AF). **(A–F)** Relative protein levels of PD-1, Claudin-1, IL-10, IL-1β and cleaved Foxp3 in the colon (*n* = 5). **(G)** Tregs related factors protein colon was detected by IHC (*n* = 3). **(H–J)** Positive area of Tregs-associated factors protein (PD-1, Foxp3 and IL-10) in the colon (*n* = 3). **(K–P)** Relative mRNA levels with β-actin of the junction proteins (Claudin-1, Occludin and ZO-1) and Tregs-associate factors protein (PD-1, Foxp3 and IL-10) in colon (*n* = 6). The data represent the mean ± SD, *p* < 0.05 was set as the threshold for significance by one-way and two-way ANOVA followed by *post hoc* comparisons using Tukey’s test for multiple groups’ comparisons. * Indicates statistical significance compared to the CON group, * *p* < 0.05, ** *p* < 0.01, # indicates statistical significance compared to the MOD group, # *p* < 0.05, ## *p* < 0.01, ^ indicates statistical significance compared to the FMT group, ^ *p* < 0.05, ^^ *p* < 0.01, +indicates statistical significance compared to the ABX group, + *p* < 0.05, ++ *p* < 0.01. WB experiment repeated more than three times, IHC experiment repeated 5 times, RT-PCR experiment repeated more than three times.

To investigate whether the protective effect of FMT against intestinal barrier damage caused by GE is associated with the modulation of the intestinal immune system, we evaluated changes in the protein expression of Tregs-related factors (Foxp3, PD-1, IL-1β, Claudin-1, IL-10) in the colon (Figures 4A–F). Compared to the CON group, a significant increase was observed in the expressions of IL-1β in the MOD and ABX groups (*p* < 0.01), following FMT, there were varying degrees of decrease in the expressions of IL-1β in the FMT group and AF group (*p* < 0.01) (Figure 4E). Compared to the CON group, a significant increase was observed in the expressions of PD-1, Foxp3, and IL-10 in the MOD and ABX groups (*p* < 0.01). Following FMT, there were varying degrees of decrease in the expressions of PD-1, Foxp3, and IL-10 in the FMT group; no significant change was observed in the AF group (Figures 4B,D,F). Similar alterations were also noted for inflammatory factor IL-1*β*. Additionally, Claudin-1 expression decreased in MOD and ABX groups (Figure 4C) but increased after FMT (*p* < 0.05).

The expression of Tregs-related protein factors in the colon was evaluated through immunohistochemistry (Figure 4G; Supplementary Table S2), and the results were consistent with those obtained from Western blot analysis (Figures 4H–J). Subsequently, we investigated tight junction proteins and Tregs-related protein factors at the mRNA level, and the expression patterns were concordant with the aforementioned findings.

### 3.5 FMT influences the composition of gut microbiota in GE rats

**Figure 5:**
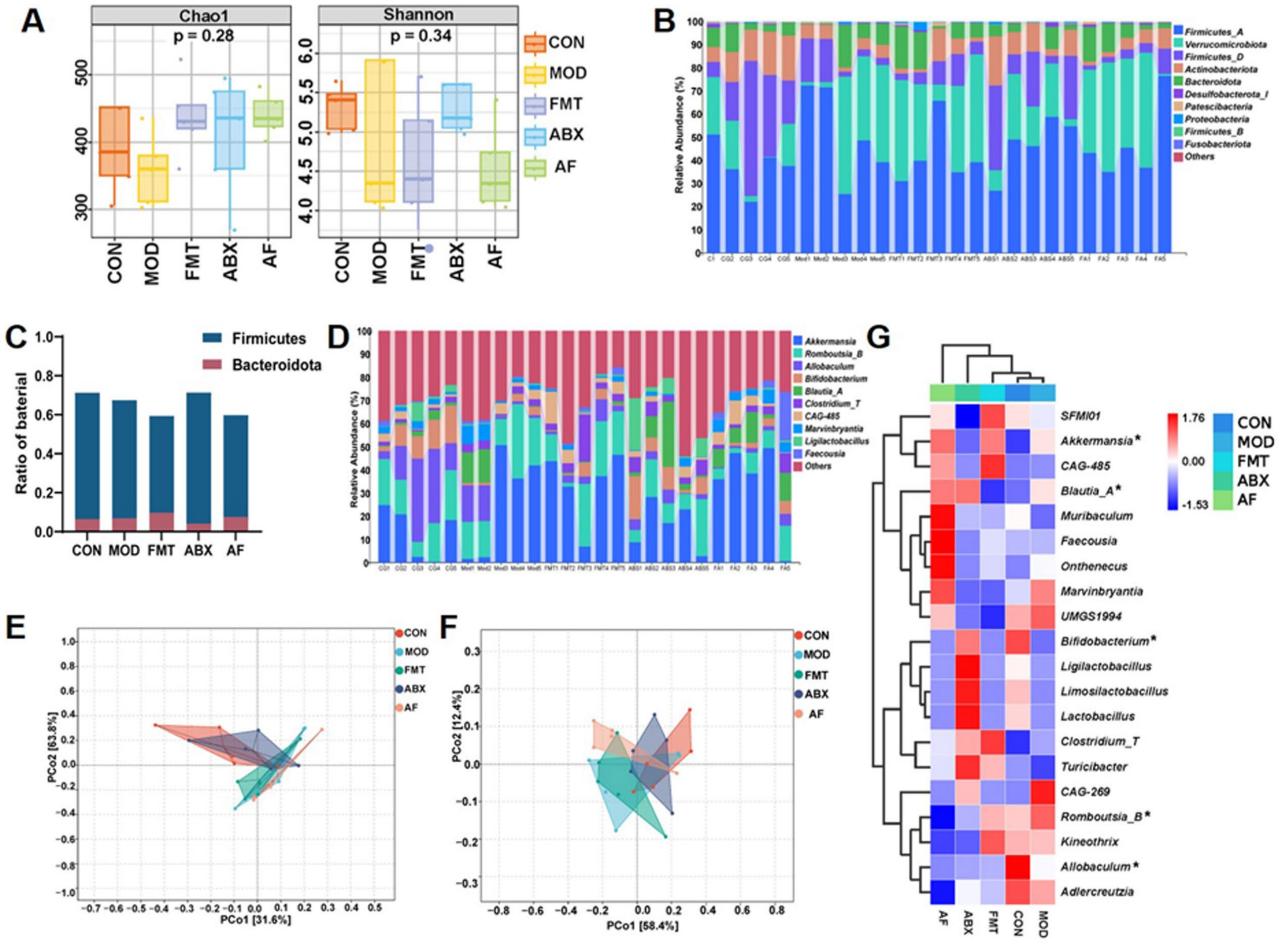
FMT intervention influences the gut microbiota composition in GE rats. The study included five groups: control group (CON), GE model group (MOD), GE+ fecal microbiota transplantation group (FMT), antibiotic clearance +GE group (ABX), and antibiotic removal +GE+ fecal microbiota transplantation group (AF). **(A)** Boxplot of alpha diversity indices (*n* = 5), the vertical axis represents the value of the corresponding alpha diversity index. **(B)** Taxonomic composition analysis at the phylum level for each sample set (*n* = 5). **(C)** Ratio of Firmicutes and Bacteroidetes in the phylum level. **(D)** Taxonomic composition analysis at the genus level for each sample set (*n* = 5). **(E)** PCA plot visualizing differences in species composition at the phylum level between samples across groups (*n* = 5). **(F)** PCA plot visualizing differences in species composition at the genus level between samples across groups (*n* = 5). **(G)** Heatmap displaying variations in species composition across groups (*n* = 5), asterisk indicate the bacterial genus with the highest enrichment at the genus level.

To assess the impact of FMT on gut microbiota modulation, we conducted 16S rRNA gene sequencing analysis to examine bacterial composition following microbial treatment in rats. A total of 25 samples from five groups of rats (*n* = 5) were utilized to generate 16S rRNA gene profiles. The ASV/OTU numbers in CON, MOD, FMT, ABX and AF groups were 750, 626, 802, 724, and 739, respectively, (Supplementary Figure S1A). When the sequencing depth exceeded 5,000 for all samples, a plateau was observed in the rarefaction curve indicating sufficient sample diversity (Supplementary Figure S1B). Additionally, when sample abundance surpassed 200, the flat trendline suggested high uniformity between samples (Supplementary Figure S1C). Alpha diversity assessment revealed no significant differences in Chao1 and Shannon indices among the five groups (*p* > 0.05) which reflect microbiota richness and diversity (Figure 5A). To evaluate community similarity, Bray-Curtis PCoA was employed for β-diversity assessment revealing that principal components PCo1, PCo2, and PCo3 explained variation at rates of 44.1, 10.41, and 8%, respectively (Figures 5E,F). At phylum level, *Firmicutes*, *Verrucomicrobiota*, and *Actinobacteriota* were predominant (Figures 5B,C). At genus level, *Akkermansia, Romboutsia_B,* and *Allobaculum* exhibited dominance (Figure 5D; Supplementary Table S3). The heatmap displays the top 20 microbial flora abundance and correlations among different groups at the genus level (Figure 5G).

**Figure 6:**
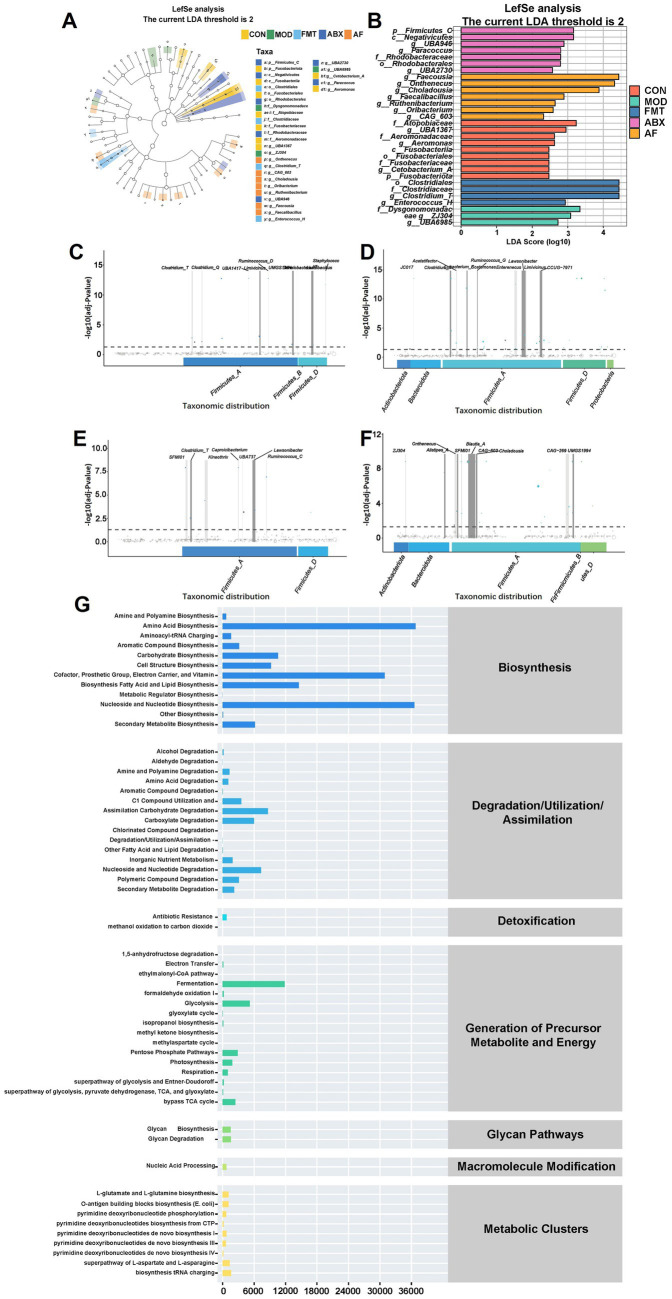
FMT modulates gut microbiota metabolite composition in GE rats. There were five groups: control group (CON), GE model group (MOD), GE+ fecal microbiota transplantation group (FMT), antibiotic clearance + GE group (ABX) and antibiotic removal + GE + fecal microbiota transplantation group (AF). **(A,B)** LDA effect size analysis of each group (*n* = 5), the vertical axis is the −log10 (adj-*P*value) value, and the more significant the difference, the higher the Y-axis position. **(C–F)** MetgenomeSeq analysis of species difference composition between sample groups (COM-MOD, CON-ABX, MOD-FMT, ABX-AF) (*n* = 5). **(G)** Statistics of metabolic pathways in bacterial genera (*n* = 5).

To identify the specific genus within each group, LEfSe and LDA were employed to pinpoint the core taxa most likely responsible for the observed differences between groups, as illustrated in Figures 6A,B. The abundance of *Firmicutes* was significantly higher in the ABX group compared to the CON group (*p* = 0.025, LDA = 3.268). The FMT group exhibited a higher abundance of *Clostridium_T* (*p* = 0.042, LDA = 4.438), while the AF group showed elevated levels of *Faecousia*, *Onthebecus*, and *Choladousin* (*Faecousia*, *p* = 0.011, LDA = 4.384; *Onthebecus*, *p* = 0.024, LDA = 4.297; *Choladousin*, *p* = 0.006, LDA = 3 0.886). Subsequently, Metagenome Seq analysis was conducted between these two groups to identify differential flora (Figures 6C–F). Compared with the CON group, the MOD group demonstrated a predominance of *Firmicutes* at genus level including *Closttridium_T* (LogFC = 3.445), *Closttridium_Q* (LogFC = 2.482), and *Lachnospiraceae* (LogFC = 3.3) (Figure 6C). In contrast, *Firmicutes* and *Proteobacteria* were dominant in the ABX group with *Closttridium_T* (LogFC = 5.404) *Ligilacttobacillus* (LogFC = 5.338) and *Eubacterium_F* (LogFC = 4.498) at the genus level (Figure 6D). Compared with MOD group, *Firmicutes* dominated in FMT group, among which *Closttridium_T* (LogFC = 4.095), *Ruminococcus_C* (LogFC = 3.908), and Lawsonibacter (LogFC = 3.277) dominated at genus level (Figure 6E). *Firmicutes* and *Bacteroidetes* were predominant in AF compared to ABX group, with *Blautia_A* (LogFC = 7.73), *Faecousia* (LogFC = 6.379), and Choladousia (LogFC = 4.877) dominating at genus level (Figure 6F). In metabolic pathway statistics, compared with the MOD group, more differential genera were enriched in amino acid biosynthesis, nucleoside, and nucleotide biosynthesis, and fatty acid and lipid biosynthesis in the FMT group (Figure 6G).

### 3.6 FMT modulates the composition of gut microbiota metabolites in genetically engineered rats

**Figure 7:**
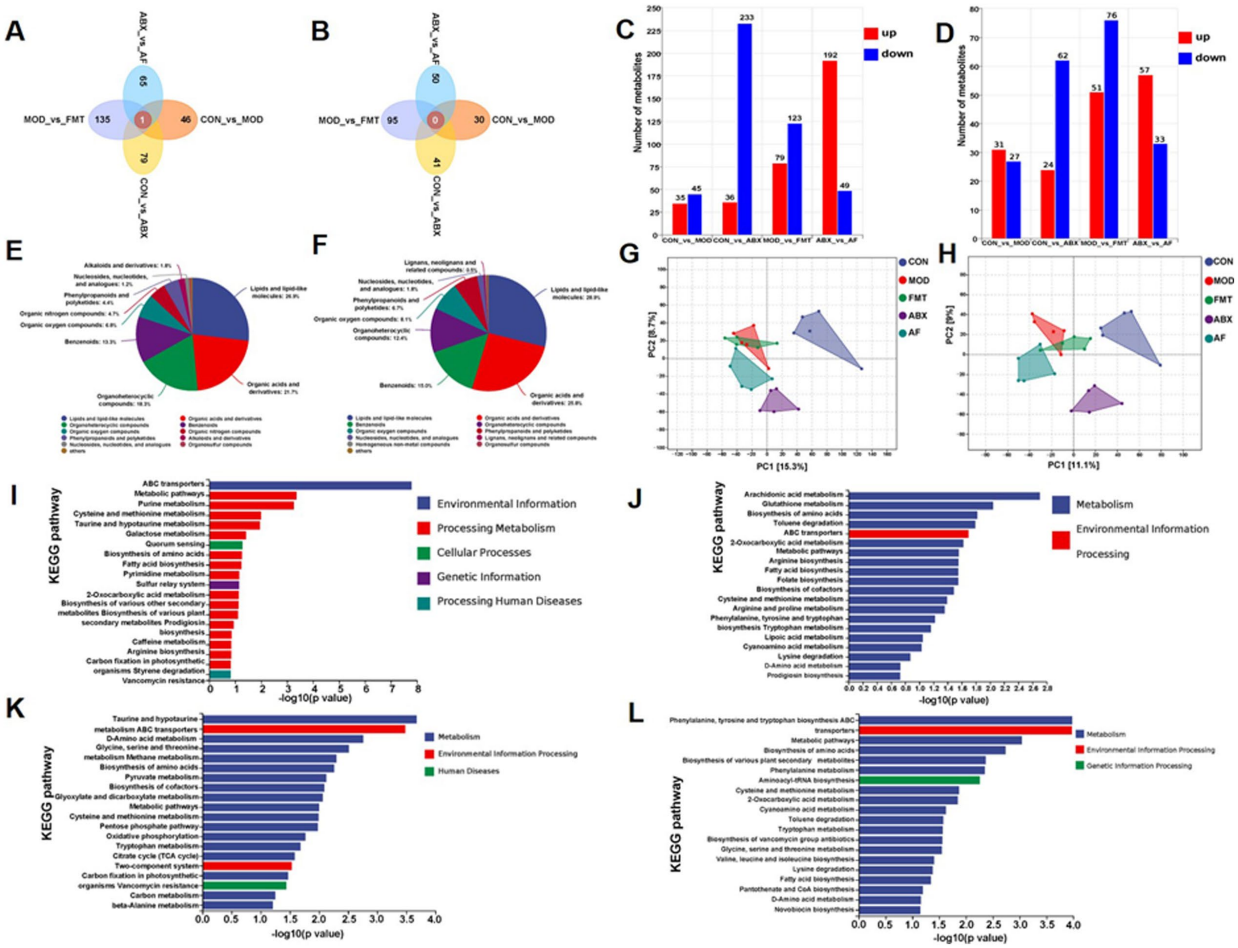
FMT modulates gut microbiota metabolite composition in GE rats. There were five groups: control group (CON), GE model group (MOD), GE+ fecal microbiota transplantation group (FMT), antibiotic clearance + GE group (ABX) and antibiotic removal + GE + fecal microbiota transplantation group (AF). **(A,B)** Venn graph of each group in positive and negative modes (*n* = 5). **(C,D)** Positive and negative modes comparison between groups of DEMs statistics (*n* = 5). **(E,F)** Positive and negative modes identification of metabolites (*n* = 5). **(G,H)** Partial Least Squares-Discriminate Analysis in positive and negative modes (*n* = 5). **(I–L)** Statistics of metabolic pathways between sample groups (COM-MOD, CON-ABX, MOD-FMT, ABX-AF) (*n* = 5).

To investigate the impact of gut microbiota on the host through metabolites, a total of 25 samples from five groups (*n* = 5) were subjected to metabolomics analysis. The Venn diagram illustrated distinct metabolic changes resulting from different treatments (Figures 7A,B). The specific number of unique metabolites in positive and negative ion modes is depicted in the figure (Figures 7C,D). A total of 1,381 metabolites were identified as significant differential metabolites based on VIP > 1 and *p* < 0.05 screening criteria. These metabolites were comprehensively categorized under positive and negative ion modes (Figures 7E,F). In order to further elucidate the distinctions among the groups, we conducted Partial Least Squares Discriminant Analysis (PLS-DA) for in-depth investigation. The PLS-DA model revealed limited separation between the five groups, with quality parameters R2X = 0.241, R2Y = 0.376, and Q2 = 0.117, indicating suboptimal reliability and predictability of the model (Figures 7J,G,H). Subsequently, KEGG enrichment analysis of differential metabolites was performed between the two groups, and the top 20 pathways exhibiting the lowest *p* value—representing the most significant enrichment—were selected for presentation (Figures 7I–L). In comparison with the CON group, the differentially expressed metabolites (DEMs) in the MOD group (Supplementary Table S4) exhibited enrichment in Cysteine and methionine metabolism, Metabolic pathways, and Purine metabolism (Figure 7I). In comparison with the CON group, the DEMs in the ABX group (Supplementary Table S5) showed enrichment in Arachidonic acid metabolism, Glutathione metabolism, and Biosynthesis of amino acids metabolic pathways (Figure 7J). Contrasted with the MOD group, the DEMs in the FMT group (Supplementary Table S6) demonstrated enrichment in Taurine and hypo taurine metabolism, Glycine, serine and threonine metabolism, as well as D-Amino acid metabolism (Figure 7K); Compared with the ABX group, The DEMs in the AF group (Supplementary Table S7) exhibited enrichment in pathways related to Phenylalanine, Tyrosine, and Tryptophan biosynthesis as well as Metabolic and amino acid biosynthesis pathways (Figure 7L).

### 3.7 Correlation between microbiome composition, differentially expressed metabolites, and phenotypic variables

**Figure 8:**
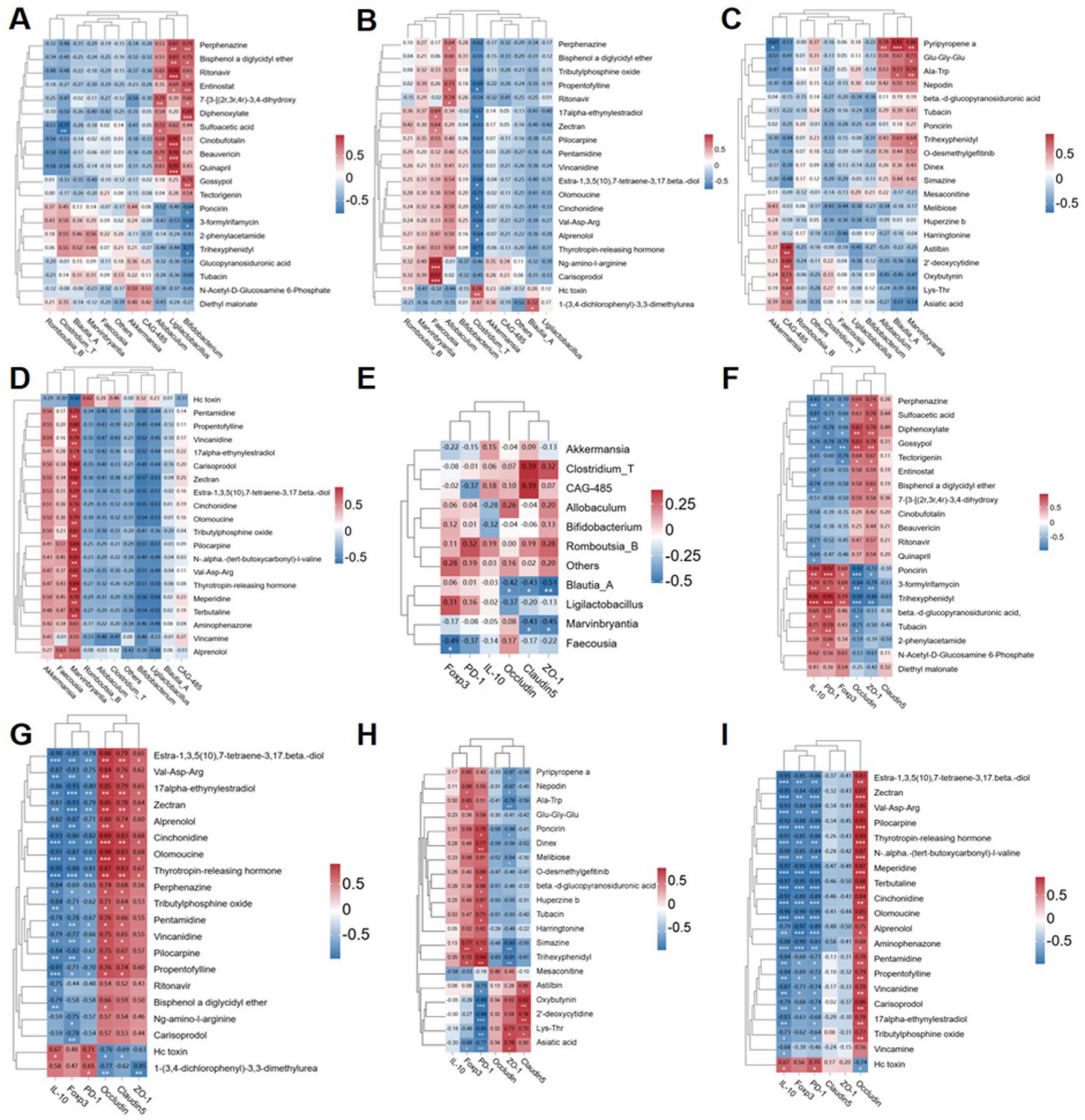
Correlation heatmap intergroup analysis was conducted to examine the relationships among microbiome composition, differentially expressed metabolites (DEMs), and phenotypic variables. **(A–D)** The top 20 DEMs were correlated with microbiome composition at the genus level in four groups (COM-MOD, CON-ABX, MOD-FMT, ABX-AF). **(E)** Correlation analysis was performed between the top 10 genera of microbiome composition and junction proteins (Claudin-1, Occludin and ZO-1), as well as Tregs-associated factors protein (PD-1, Foxp3 and IL-10) in the striatum. **(F–I)** Additionally, correlation analysis was carried out between the top 20 DEMs in the four groups and junction proteins (Claudin-1, Occludin and ZO-1), along with Tregs-associated factors protein (PD-1, Foxp3 and IL-10) in the striatum.

In order to further investigate the mutual influence of microbiome composition, differentially expressed metabolites (DEMs), and phenotypic variables, we conducted pairwise correlation analysis involving two to three variables. Additionally, we performed correlation analysis to explore the interaction among microbiome composition, DEMs, and phenotypic variables through pairwise comparison of the three. We focused on the top 10 bacterial genera with the most significant differences at the genus level (Supplementary Table S3). The top 20 metabolites exhibiting the largest differences in various comparison groups (MOD vs. CON: Supplementary Table S4; ABX vs. CON: Supplementary Table S5; FMT vs. MOD: Supplementary Table S6; AF vs. ABX: Supplementary Table S7) were selected for correlation analysis (Figures 8A–D). In comparison with the CON group, *Allobaculum* and *Ligilactobacillus* in the MOD group exhibited significant correlations with differentially expressed metabolites (DEMs) (Figure 8A). In comparison with the CON group, *Allobaculum*, *Clostridium_T*, etc., displayed correlations with DEMs in the ABX group (Figure 8B). When compared to the MOD group, *Marvinbryantia* and *Blautia_A* demonstrated correlations with DEMs in the FMT group (Figure 8C). Conversely, compared to the ABX group, *Marvinbryantia*, *Faecousia*, etc., exhibited correlation with DEMs in the AF group (Figure 8D). The microbiota did not exhibit strong correlations with metabolites; therefore, we conducted a correlation analysis between the microbiota and tight junction proteins as well as Tregs-related factors (Figure 8E). Our findings revealed a positive correlation between the expression of tight junction proteins and *Ligilactobacillus* and *Blautia_A*. Conversely, Tregs-related factors exhibited a negative correlation with *Ligilactobacillus* and *Allobaculum*. Additionally, we examined the correlation between differentially expressed genes (DEGs) and the expression of brain barrier function indexes (Occludin, Claudin-1, and ZO-1) and immune function indexes (IL-10, Foxp3, and PD-1) (Figures 8F–I). The results indicated that, in comparison with the CON group, the MOD group exhibited positive correlations between poncirin and trihexyphenidyl, among others, and the index expression. Conversely, perphenazine and sulfoacetic acid showed negative correlations with the index expression (Figure 8F). In the ABX group, hc toxin displayed a positive correlation while zectran and cinchonidine, among others, exhibited negative correlations (Figure 8G). Furthermore, compared to the MOD group, the FMT group demonstrated positive correlations for trihexyphenidyl and simazine among others; however, oxybutynin showed a negative correlation (Figure 8H). Lastly, in comparison with the ABX group, hc toxin was positively correlated with the brain barrier function indexes (Occludin, Claudin-1, and ZO-1) and immune function indexes (IL-10, Foxp3, and PD-1) in the AF group, while zectran and pilocapine were negatively correlated with those aforementioned indexes (Figure 8I).

## 4 Discussion

Numerous studies have highlighted the significant role of the “gut-brain axis” in mediating communication between gut and brain tissues, with the amelioration of traumatic brain injury (TBI) being closely associated with the modulation of intestinal flora (Liu et al., 2020). Conversely, dysbiosis of intestinal flora may result in compromised brain function, this type of injury can lead to neuroinflammation and other injuries (Alam et al., 2018), and various pro-inflammatory factors can cause chronic neuroinflammatory symptoms, leading to cognitive impairment and disturbances in memory formation (Kim et al., 2021; Kim et al., 2020). Hence, we postulate that enhancing gut microbiota could mitigate gas explosion (GE) induced cognitive impairment by modulating brain tissue via the “gut-brain axis”.

To investigate this hypothesis, we developed both a GE model and an FMT model to further validate the role of gut microbiota in cognitive impairment induced by GE. Additionally, we conducted an antibiotic clearance experiment to deplete the majority of gut microbiota and facilitate bacterial colonization (Jing et al., 2021). Our findings indicate that total moving distance and average moving speed in the open field box were restored in both FMT and AF rats following fecal bacteria transplantation from the CON group into recipient rats, suggesting that microbial dysbiosis caused by GE may lead to cognitive impairment. Furthermore, we observed a reduction in brain tissue cell count, mitochondrial cavitation, and loss of most cristae in MOD and ABX rats, all of which showed improvement after FMT treatment, consistent with recent research (Wang X. et al., 2023). Similarly, we observed that GE led to the down-regulation of tight junction proteins (Claudin-1, Occludin, and ZO-1) and the up-regulation of Tregs-related factors protein (IL-10, PD-1, and Foxp3), as well as pro-inflammatory cytokines (IL-1β) in the colon and striatum of MOD and ABX rats. Following the remodeling of intestinal flora in FMT experiment, all indices were restored. These findings indicate that GE induces both damage and inflammation in both the intestinal barrier and brain barrier of rats, which contributes to cognitive impairment and subsequent immune system activation. We posit that modulating intestinal flora directly contributes to ameliorating colon injury while working in conjunction with the immune system to facilitate repair of the blood–brain barrier and nervous system via the “gut-brain axis”, thereby enhancing cognitive function.

The alpha diversity of the gut microbiota decreased after GE, indicating a reduction in species richness. In mammals, the decrease in gut bacterial species has been linked to an elevated risk of cognitive dysfunction through the “gut-brain axis” (Zheng et al., 2024). Furthermore, GE led to significant structural changes in the gut bacterial community of male rats, characterized by increased abundance of *Akkermansia*, *Romboutsia* and *Allobaculum* at the genus level, consistent with our previous findings (Dong et al., 2024). The gut microbiota of recipient rats following FMT exhibited similar behavior to that of the Control group. *Akkermansia* is a gram-negative anaerobic bacterium representing *Verrucomicrobia* and plays a key role as a mucin-degrading bacterium in the colon (Cani et al., 2022). Studies have indicated that *Akkermansia* may modulate TBI in the cerebral cortex by regulating NLRP3 inflammatory activation to ameliorate nerve inflammation and neurological damage (Chen et al., 2023). Furthermore, it can also restore mitochondrial structure and function to mitigate brain injury and intestinal barrier dysfunction by modulating mitochondrial homeostasis and metabolism (Lian et al., 2023). This aligns with our observations from H&E staining and TEM analysis. Additionally, *Akkermansia* is capable of producing butyric acid in the intestine (DeSana et al., 2024), which has been reported in studies to improve dopamine metabolism disorders and cognitive impairment (Wang et al., 2020). Meanwhile, studies have shown that with the enrichment of *Akkermansia*, the concentration of SCFAs also increases (Li et al., 2023), this also indicates the positive role of *Akkermansia* in the intestine. Current research suggests that butyric acid, as an intestinal metabolite, is among the most crucial short-chain fatty acids for TBI repair (Witkin et al., 2024). As a newly discovered genus, recent studies have indicated that an decrease in *Allobaculum* abundance in the colon may contribute to brain injury recovery (Yang et al., 2023), enhance the level of gut brain barrier proteins and alleviate brain inflammation response (Yuan et al., 2022). There is also study indicating that as a gut probiotic, *Allobaculum* has the potential to reshape the gut brain axis as a mediator and repair nerve injury (Rahman et al., 2023). The two types of bacteria, which are the intestinal bacteria in the intestinal mucosa, play distinct roles in the composition and integrity of the intestinal barrier (van Muijlwijk et al., 2021). It is noteworthy that *C. butyricum* was also found to be one of the most abundant bacteria in the microbiota. Numerous studies have confirmed that *C. butyricum* metabolizes butyrate in the colon and exerts its effects on brain tissue through the “gut-brain axis” (Stoeva et al., 2021), *C. butyricum* can improve the integrity of the blood–brain barrier (Liu et al., 2024) and thus improve cognitive impairment (Wang J. et al., 2023). The three species mentioned above may contribute to our experimental results and validate our hypothesis. They recolonize the colon following FMT, ameliorating intestinal injury and TBI, consequently enhancing cognitive ability. Interestingly, LefSe analysis revealed an increase in relative abundance of *g_Clostridia_T* in both FMT and AF groups after FMT. Furthermore, elevated levels of *g_Faecalibacillus* and *g_Oribacterium* identified in our study were found to activate the immune system (Wang et al., 2024). Subsequently, we identified enriched metabolic pathways at the genus level of gut microbiota. The top three pathways enriched in bacterial genera were amino acid biosynthesis, nucleoside and nucleotide biosynthesis, and fatty acid and lipid biosynthesis. Previous studies have indicated that fatty acid biosynthesis can dynamically improve neurodegeneration and nerve regeneration, as well as help to ameliorate cognitive decline caused by TBI (Witkin et al., 2024). Meanwhile, nucleoside and nucleotide biosynthesis pathways have been associated with restoring hippocampal function and preventing or ameliorating schizophrenia-like behaviors (Hao et al., 2022). In addition, fatty acid and lipid biosynthesis play a crucial role in maintaining lipid balance in the body. Their oxidative stress can alleviate nerve inflammation and neurodegeneration by activating astrocytes and stimulating neurons through the IL-3 signaling pathways (Mi et al., 2023). The significant involvement of gut microbiota in CNS disease is undeniable. Conversely, gut microbes have the potential to reverse brain function deterioration. For instance, transplanting fecal microbes from young mice into older mice can delay aging-induced impairments in brain function, including cognitive-behavioral deficits (D'Amato et al., 2020). These findings underscore the substantial therapeutic potential for reversing TBI-related effects by modulating the gut microbiota (Choi and Cho, 2016). Therefore, it would be valuable to observe the behavioral phenotypes of rats treated with FMT in future studies to explore its exceptional therapeutic potential. Our study demonstrates that GE-induced gut microbiota disorder could contribute to increased proinflammatory cytokines in the brain. These results suggest that modulating the gut microbiota could serve as a therapeutic target for GE-induced TBI. For example, optimizing the gut microbiota through probiotic administration and FMT may be a prospective strategy for treating GE-induced TBI and subsequent long-term cognitive dysfunction.

In animal models, glyphosate exposure can induce chronic gut and systemic inflammation, often accompanied by dysbiosis of the gut microbiota (Dong et al., 2024). Our findings indicate that normal rats exposed to glyphosate may experience more severe inflammation than control rats, suggesting a potential exacerbation of inflammation by gut bacteria. The permeability of the gut epithelium is primarily regulated by a series of tight junction (TJ) proteins expressed by gut epithelial cells. Proinflammatory cytokines such as TNF-*α*, IFN-*γ*, and IL-1β can disrupt intestinal TJ proteins, leading to increased permeability of the gut epithelium (Al-Sadi et al., 2009). TJ proteins restrict paracellular movement of molecules between adjacent epithelial cells and form a network regulatory mechanism that anchors neighboring cells while maintaining the physical barrier of the gut. ZO-1 belongs to a group of auxiliary TJ proteins that bind Occludin, a transmembrane TJ protein, to the cytoskeleton (Huang et al., 2020). The protein Claudin-1 is responsible for maintaining the integrity of the colon mucosal barrier and ensuring its functional stability (Huang et al., 2020). Our findings indicate that rats exposed to GE showed reduced expression of TJ proteins, which are crucial for protecting epithelial cells. These results suggest an increase in colon permeability in rats following exposure to GE. Furthermore, FMT confirmed that gut microbiota disorder induced by GE leads to gut barrier damage, potentially facilitating bacterial translocation.

To further investigate the potential mechanism of gut microbiota in ameliorating cognitive impairment induced by GE, we conducted untargeted metabolomics to monitor alterations in metabolites. We observed distinct changes in metabolites among different groups, mirroring microbial shifts. Following FMT treatment of GE model rats, we were surprised to discover an enrichment of more DEMs in Taurine and hypo taurine metabolism. Intestinal injury results in taurine metabolism dysregulation and inflammatory response, leading to intestinal barrier damage (Zhou et al., 2019). Studies have shown that taurine can mitigate inflammation and oxidative stress via the MAPK signaling pathway to alleviate LPS-induced injury (Zhang B. et al., 2022). LPS has been demonstrated to induce an inflammatory response by enhancing the permeability of the blood–brain barrier and translocating across the intestinal barrier into systemic circulation (Galea, 2021). LPS is recognized as a significant factor in disrupting the BBB, which restricts the entry of molecules from systemic circulation into brain tissue and is essential for maintaining CNS stability (Banks and Robinson, 2010). Elevated levels of LPS in brain circulation led to increased BBB permeability and subsequent brain damage. The BBB is a highly specialized structure formed by brain micro vessels through TJ proteins, such as ZO-1 and Occludin, that link adjacent cells (Cardoso et al., 2010). Reduced expression or altered distribution of TJ proteins in brain endothelium can result in BBB impairment, leading to neurovirulence, neuroinflammation, and potentially contributing to cognitive dysfunction. Our study revealed that gut microbiota imbalance induced by GE serves as a source of inflammatory response, potentially leading to elevated proinflammatory cytokines in the brain and reduced levels of brain TJ proteins.

Furthermore, a significant number of differentially expressed metabolites (DEMs) are involved in D-amino acid metabolism. Research has also demonstrated that the release of D-serine from glial cells can inhibit synaptic damage, restore nerve cell function, and consequently improve cognitive function (Tapanes et al., 2022). In this study, more metabolites were enriched in these two pathways, supporting our hypothesis that activating these pathways may improve cognitive impairment caused by GE via the “gut-brain axis.” Importantly, fatty acid biosynthesis was also a key focus of DEMs enrichment. This pathway primarily generates new fatty acids in the gut, while SCFAs are products of intestinal flora and their levels are inversely related to inflammation (Opeyemi et al., 2021). Thus, the metabolite pathway enrichment results were consistent with our microbiome experiments, further confirming the importance of the “gut-brain axis” in improving cognitive impairment. Gut bacteria play a significant role in this process. Additionally, Person correlation analysis revealed that the microbiota and DEMs enriched in these pathways correlated with the expression of tight junction proteins (ZO-1, Claudin-5, and Occludin) and Tregs-related factors (IL-10, PD-1, and Foxp3). These findings suggest that GE may induce inflammatory responses, which could be exacerbated by gut bacteria. Furthermore, FMT demonstrated that GE-induced gut microbiota dysbiosis and metabolic disorders could trigger increased brain proinflammatory cytokines. Therefore, it is important to consider that while gut bacteria are one cause of GE-induced TBI, they are not the only cause (Dong et al., 2020). Furthermore, the LPS-induced inflammatory response can result in abnormal behavior. For instance, brain inflammation triggered by LPS may contribute to GE-induced TBI development (Dong et al., 2024). however, behavioral changes (e.g., exploratory activity) are not always linked to brain inflammation (Fragopoulou et al., 2019). Therefore, behavioral experiments would help to extend our findings. In future studies on GE injury, more research should be performed on the relationship between gut microbiota, brain inflammation, and behavioral phenotypes.

Our research has provided new insights into understanding the injury mechanism of GE-induced TBI at a deeper level. However, while we used molecular experimental data to characterize brain inflammation, we did not conduct functional analysis. As a result, our comprehensive characterization of specific damage to the brain and barrier function was limited. Although FMT confirmed that GE-induced gut microbiota disorder may contribute to brain inflammation through the fatty acid biosynthesis pathway, it remains uncertain whether this is the sole contributing factor. We recommend additional intervention approaches such as cytokine antibody neutralization and *in vitro* experiments to confirm this association.

## 5 Conclusion

**Figure 9:**
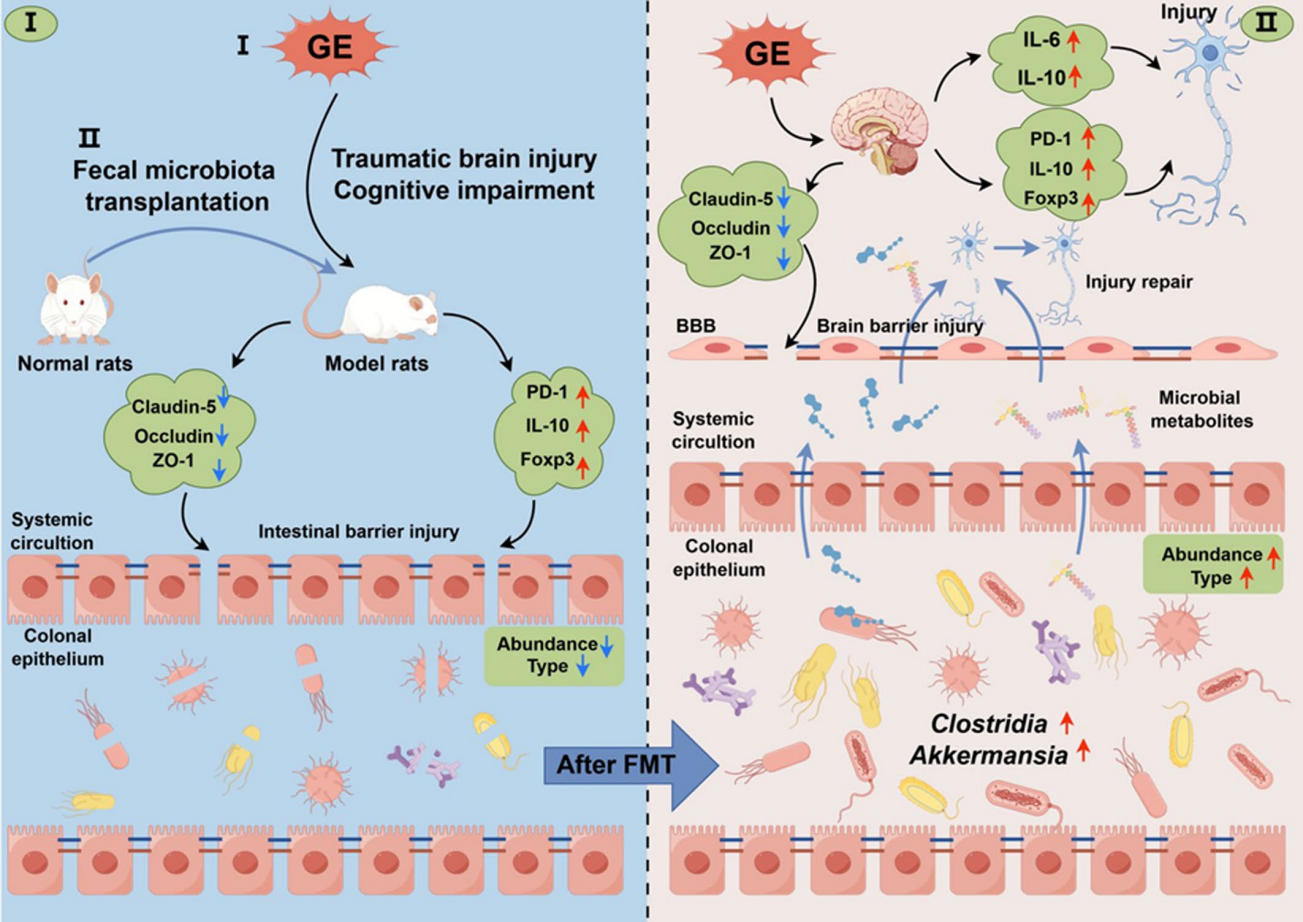
Schematic representation of fecal microbiota transplantation (FMT) intervention in the context of colon and brain barrier function injury/repair following traumatic brain injury (TBI) induced by GE (GE). GE induces dysbiosis of the abundance and type of gut microbiota in male TBI rats. Targeted supplementation of gut bacteria not only mitigated acute brain injury and cognitive dysfunction, but also significantly improved the immune microenvironment, the types and abundance of gut microbiota are increased after FMT, facilitating restoration of colon and brain barrier function after GE-induced TBI. These findings were corroborated by the application of FMT intervention post-TBI. Collectively, these results demonstrate that gut microbiota and their metabolites promote restorative activation of the “gut-brain axis” for neuroprotection, contributing to accelerated repair of colon and brain barriers as well as recovery of cognitive function after GE-induced TBI.

Gas explosion increases the risk of traumatic brain injury in miners. Currently, research on the pathogenic mechanism of GE-induced TBI is limited. However, our study provides new insights into addressing these unknowns. In summary, GE triggers dysbiosis of intestinal flora in male TBI rats. Targeted supplementation of gut bacteria not only reduced acute brain injury and cognitive dysfunction but also significantly improved the immune microenvironment, promoting restoration of colon and brain tight junctions after GE-induced TBI. These findings were further supported by administering an FMT intervention post-TBI. Collectively, these results demonstrate that specific gut microbiota such as *Clostridium_T* and *Allobaculum*, along with their metabolites, stimulate restorative “gut-brain axis” activation for neuroprotection, contributing to accelerated repair of the colon and brain as well as recovery of cognitive function after GE-induced TBI (Figure 9).

## Data Availability

The datasets presented in this study can be found in online repositories. The names of the repository/repositories and accession number(s) can be found in the article/Supplementary material.

## Footnotes

^1^https://docs.qiime2.org/2022.11/tutorials/

^2^https://metacyc.org/

^3^https://www.kegg.jp/
