# Supplementary material for: Fecal microbiota transplantation alleviates cognitive impairment by improving gut microbiome composition and barrier function in male rats of traumatic brain injury following gas explosion
Source: Front Microbiol. 2024 Nov 1;15:1485936. doi: 10.3389/fmicb.2024.1485936 (PMC11564976; doi:10.3389/fmicb.2024.1485936)
Supplement: Supplementary file 1 [file Data_Sheet_1.docx]

**Supplemental Figure**


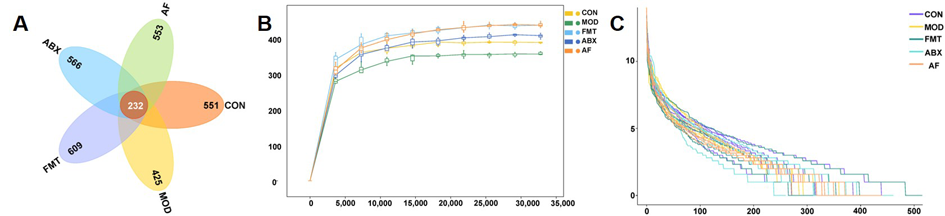


Fig. S1 ASV/OTU values (A) and richness of gut microbiota (B and C) in each group. (A) Venn diagram depicting the overlap of microbial species in each group (n=5). (B) Rarefaction curves illustrating species richness in each group (n=5). (C) Rank abundance curves showing species evenness in each group (n=5). Note: ASV, Amplicon Sequence Variant; OUT, Operational Taxonomic Units
