# Supplementary material for: Fecal microbiota transplantation alleviates cognitive impairment by improving gut microbiome composition and barrier function in male rats of traumatic brain injury following gas explosion
Source: Front Microbiol. 2024 Nov 1;15:1485936. doi: 10.3389/fmicb.2024.1485936 (PMC11564976; doi:10.3389/fmicb.2024.1485936)
Supplement: Supplementary file 2 [file Table_1.docx]

Table S1 Primer names and primer sequences for mRNA detection by RT-PCR.

| Primer name | Sequence (5' to 3') |
| --- | --- |
| β-actin | Forward: CACGATGGAGGGGCCGGACTCATC |
|  | Reverse: TAAAGACCTCTATGCCAACACAGT |
| IL-10 | Forward: GTCCTTTCACTTGCCCTCATC |
|  | Reverse: CAAACTGGTCACAGCTTTCGA |
| Occludin | Forward: ATAGCCATTGTCCTGGGGTTCAT |
|  | Reverse: TCCATCTTTCTTCGGGTTTTCAC |
| ZO-1 | Forward: TGATCGTCTGTCCTACCTGTC |
|  | Reverse: CGCCTTCTGTATCTGTGTCTT |
| Claudin-1 | Forward: GAGACTACCACTGTCCCC |
|  | Reverse: AAAGAATCCTCAAAACCA |
| Foxp3 | Forward: GGCTCTACTCTGCACCTTCC |
|  | Reverse: GCAGTGGGTAGGATCCTTGT |
| Claudin-5 | Forward: GGGTCTGGTGCTGTGTCTGGTA |
|  | Reverse: TTCATACACCTTGCACTGCATG |
| PD-1 | Forward: GCTGGTAGCAAACTCCTG |
|  | Reverse: AGACTCCTATCTGCCTCACT |

Table S2 Raw data for the ratio of positive area of colon tissue for PD-1, Foxp3 and IL-10.

| PD-1 | | | | |  | Foxp3 | | | | |  | IL-10 | | | | |
| --- | --- | --- | --- | --- | --- | --- | --- | --- | --- | --- | --- | --- | --- | --- | --- | --- |
| CON | MOD | FMT | ABX | AF |  | CON | MOD | FMT | ABX | AF |  | CON | MOD | FMT | ABX | AF |
| 4.05 | 6.88 | 5.71 | 7.01 | 6.17 |  | 14.75 | 24.98 | 16.41 | 38.25 | 24.14 |  | 17.94 | 34.8 | 9.37 | 42 | 26.93 |
| 2.15 | 5.86 | 5.41 | 7.84 | 5.95 |  | 14.24 | 21.48 | 14.42 | 29.36 | 15.89 |  | 20.07 | 22.11 | 20.23 | 36 | 39.73 |
| 2.51 | 8.82 | 2.85 | 8.89 | 5.18 |  | 11.83 | 20.57 | 11.95 | 26.58 | 23.96 |  | 18.23 | 13.81 | 11.46 | 38.54 | 33.56 |
| 3.07 | 8.98 | 4.56 | 8.51 | 5.47 |  | 12.56 | 23.08 | 14.75 | 39.93 | 24.04 |  | 22.56 | 18.45 | 10.85 | 34.25 | 38.67 |
| 3.93 | 8.66 | 5.93 | 7.66 | 5.77 |  | 13.78 | 20.46 | 14.24 | 34.1 | 22.74 |  | 17.68 | 21.22 | 20.34 | 26.93 | 20.78 |

Table S3 Top 10 differences flora in average proportion of each group of genus level.

| ID | Mean CON | Mean MOD | Mean FMT | Mean ABX | Mean AF |
| --- | --- | --- | --- | --- | --- |
| *Akkermansia* | 0.134022392 | 0.266397026 | 0.335164718 | 0.160134808 | 0.343747768 |
| *Romboutsia_B* | 0.159084502 | 0.190728384 | 0.165545364 | 0.122603528 | 0.077309776 |
| *Allobaculum* | 0.19516537 | 0.069315508 | 0.039098748 | 0.033297046 | 0.020862932 |
| *Bifidobacterium* | 0.098763934 | 0.017487092 | 0.023493816 | 0.08576179 | 0.02518634 |
| *Blautia_A* | 0.01892058 | 0.057142226 | 0.00573832 | 0.084972268 | 0.083562172 |
| *Clostridium_T* | 0.01390977 | 0.028201896 | 0.06614074 | 0.050493312 | 0.03556902 |
| *CAG-485* | 0.019761576 | 0.021605406 | 0.054714212 | 0.021469678 | 0.042916244 |
| *Marvinbryantia* | 0.02290699 | 0.038490818 | 0.01248369 | 0.021469678 | 0.043992602 |
| *Ligilactobacillus* | 0.026377014 | 0.004231776 | 0.00436239 | 0.021469678 | 0.003826698 |
| *Faecousia* | 0.007066288 | 0.006945916 | 0.011979538 | 0.021469678 | 0.05523382 |

Table S4 Top 20 DEMs in positive and negative ion modes were screened between MOD and CON groups using metabonomics.

| ID | Name | VIP | m/z | Regulation | Mean CON | Mean MOD | log2FoldChange | *p*value |
| --- | --- | --- | --- | --- | --- | --- | --- | --- |
| M404T248.pos | Perphenazine | 2.891080413 | 404.15276 | Down | 127131793.1 | 778533.0216 | -7.351350917 | 1.22E-06 |
| M593T366.neg | Poncirin | 2.706898086 | 593.18763 | Up | 44434.49751 | 2947585.099 | 6.051709482 | 1.12E-05 |
| M499T375.neg | Gossypol | 2.645359134 | 499.16726 | Down | 14114037.66 | 257158.8242 | -5.778327295 | 6.20E-05 |
| M361T274.pos | Ritonavir | 2.48521929 | 361.14702 | Down | 22490675.81 | 672095.1519 | -5.064517705 | 0.005962843 |
| M377T169_1.pos | Entinostat | 2.23866523 | 377.17861 | Down | 89939959.17 | 6521343.11 | -3.785721197 | 0.009632208 |
| M381T448.pos | Cinobufotalin | 2.198640524 | 381.1871 | Down | 43900317.89 | 3768955.036 | -3.541994898 | 0.019506941 |
| M302T264_2.pos | Trihexyphenidyl | 2.87044607 | 302.24379 | Up | 1723795.639 | 20023375.98 | 3.538024582 | 6.23E-06 |
| M363T246_1.pos | Bisphenol a diglycidyl ether | 2.451515299 | 363.16279 | Down | 76847485.6 | 6919410.831 | -3.47327695 | 0.002710563 |
| M586T325.pos | Glucopyranosiduronic acid | 2.100959474 | 586.18773 | Up | 4291966.077 | 42364874.98 | 3.303158033 | 0.02497904 |
| M453T475.pos | Diphenoxylate | 2.45965437 | 453.24453 | Down | 4244903.78 | 472780.1283 | -3.166490549 | 0.003697974 |
| M461T364.pos | Quinapril | 2.310787118 | 461.21064 | Down | 9145305.653 | 1021706.015 | -3.162051253 | 0.005967474 |
| M136T392.pos | 2-phenylacetamide | 2.094374681 | 136.09691 | Up | 9262167.163 | 81808896.53 | 3.142836041 | 0.033088924 |
| M284T85.neg | Tectorigenin | 1.925161666 | 284.0269 | Down | 163701272.2 | 19238323.45 | -3.08901055 | 0.026722659 |
| M300T250.neg | Glucosamine 6 | 1.814736698 | 300.03959 | Up | 76270283.23 | 519076104.5 | 2.766753113 | 0.041522186 |
| M585T441.pos | Dihydroxy | 2.09350371 | 585.14314 | Down | 7404652.986 | 1190354.422 | -2.637040934 | 0.044171403 |
| M139T22.neg | Sulfoacetic acid | 2.299331522 | 138.98594 | Down | 6168473.489 | 1030265.13 | -2.581897859 | 0.005616049 |
| M806T441.pos | Beauvericin | 1.977014761 | 806.41658 | Down | 3817281.767 | 643765.4596 | -2.567938603 | 0.046025242 |
| M720T390.neg | Tubacin | 1.906977894 | 720.26539 | Up | 360248.6707 | 2126694.057 | 2.561547492 | 0.027977828 |
| M133T307.pos | Diethyl malonate | 2.137671176 | 133.06081 | Up | 1831359.017 | 10778232.29 | 2.557134037 | 0.026755143 |
| M724T353.neg | 3-formylrifamycin | 1.898291941 | 724.31534 | Up | 25071259.67 | 140005706.1 | 2.481379238 | 0.045953026 |

Note: DEMs: differently expression metabolites; VIP: Variable Importance in Projection; CON: Control group; MOD: Gas explosion model group; pos: positive ion mode; neg: negative ion mode.

Table S5 Top 20 DEMs in positive and negative ion modes were screened between ABX and CON groups using metabonomics.

| ID | Name | VIP | m/z | Regulation | Mean CON | Mean ABX | log2FoldChange | *p*value |
| --- | --- | --- | --- | --- | --- | --- | --- | --- |
| M404T248.pos | Perphenazine | 2.89108041 | 404.1528 | Down | 127131793 | 778533.022 | -7.351350917 | 1.22E-06 |
| M593T366.neg | Poncirin | 2.70689809 | 593.1876 | Up | 44434.4975 | 2947585.1 | 6.051709482 | 1.12E-05 |
| M499T375.neg | Gossypol | 2.64535913 | 499.1673 | Down | 14114037.7 | 257158.824 | -5.778327295 | 6.20E-05 |
| M361T274.pos | Ritonavir | 2.48521929 | 361.147 | Down | 22490675.8 | 672095.152 | -5.064517705 | 0.00596284 |
| M377T169_1.pos | Entinostat | 2.23866523 | 377.1786 | Down | 89939959.2 | 6521343.11 | -3.785721197 | 0.00963221 |
| M381T448.pos | Cinobufotalin | 2.19864052 | 381.1871 | Down | 43900317.9 | 3768955.04 | -3.541994898 | 0.01950694 |
| M302T264_2.pos | Trihexyphenidyl | 2.87044607 | 302.2438 | Up | 1723795.64 | 20023376 | 3.538024582 | 6.23E-06 |
| M363T246_1.pos | Bisphenol a diglycidyl ether | 2.4515153 | 363.1628 | Down | 76847485.6 | 6919410.83 | -3.47327695 | 0.00271056 |
| M586T325.pos | Glucopyranosiduronic acid | 2.10095947 | 586.1877 | Up | 4291966.08 | 42364875 | 3.303158033 | 0.02497904 |
| M453T475.pos | Diphenoxylate | 2.45965437 | 453.2445 | Down | 4244903.78 | 472780.128 | -3.166490549 | 0.00369797 |
| M461T364.pos | Quinapril | 2.31078712 | 461.2106 | Down | 9145305.65 | 1021706.02 | -3.162051253 | 0.00596747 |
| M136T392.pos | 2-phenylacetamide | 2.09437468 | 136.0969 | Up | 9262167.16 | 81808896.5 | 3.142836041 | 0.03308892 |
| M284T85.neg | Tectorigenin | 1.92516167 | 284.0269 | Down | 163701272 | 19238323.5 | -3.08901055 | 0.02672266 |
| M300T250.neg | Glucosamine 6 | 1.8147367 | 300.0396 | Up | 76270283.2 | 519076105 | 2.766753113 | 0.04152219 |
| M585T441.pos | Dihydroxy-4 | 2.09350371 | 585.1431 | Down | 7404652.99 | 1190354.42 | -2.637040934 | 0.0441714 |
| M139T22.neg | Sulfoacetic acid | 2.29933152 | 138.9859 | Down | 6168473.49 | 1030265.13 | -2.581897859 | 0.00561605 |
| M806T441.pos | Beauvericin | 1.97701476 | 806.4166 | Down | 3817281.77 | 643765.46 | -2.567938603 | 0.04602524 |
| M720T390.neg | Tubacin | 1.90697789 | 720.2654 | Up | 360248.671 | 2126694.06 | 2.561547492 | 0.02797783 |
| M133T307.pos | Diethyl malonate | 2.13767118 | 133.0608 | Up | 1831359.02 | 10778232.3 | 2.557134037 | 0.02675514 |
| M724T353.neg | 3-formylrifamycin | 1.89829194 | 724.3153 | Up | 25071259.7 | 140005706 | 2.481379238 | 0.04595303 |

Note: DEMs: differently expression metabolites; VIP: Variable Importance in Projection; CON: Control group; ABX: Antibiotic clearance + gas explosion model group; pos: positive ion mode; neg: negative ion mode.

Table S6 Top 20 DEMs in positive and negative ion modes were screened between FMT and MOD groups using metabonomics.

| ID | Name | VIP | m/z | Regulation | Mean MOD | Mean FMT | log2FoldChange | *p*value |
| --- | --- | --- | --- | --- | --- | --- | --- | --- |
| M431T285.neg | O-desmethylgefitinib | 1.84642567 | 431.1346 | Down | 15548723.8 | 211013.723 | -6.203315532 | 0.01749675 |
| M720T390.neg | Tubacin | 2.02442764 | 720.2654 | Down | 2126694.06 | 147222.397 | -3.85254343 | 0.00143572 |
| M302T264_2.pos | Trihexyphenidyl | 2.39565626 | 302.2438 | Down | 20023376 | 1575568.09 | -3.667741229 | 6.42E-06 |
| M584T450.pos | Pyripyropene a | 2.13295962 | 584.2303 | Down | 8412683.93 | 767425.082 | -3.45446832 | 0.00469213 |
| M267T374.pos | Dinex | 1.9426584 | 267.1077 | Down | 84854147.4 | 9299120.38 | -3.189819015 | 0.01145515 |
| M632T419.pos | Mesaconitine | 1.5150239 | 632.283 | Up | 1160213.69 | 8504941.9 | 2.87391083 | 0.04959574 |
| M451T318.pos | Astilbin | 1.67072384 | 451.1424 | Up | 5005862.21 | 32832892.6 | 2.713451371 | 0.04691846 |
| M240T257_1.pos | Huperzine b | 2.14656059 | 240.1343 | Down | 81564573.9 | 13471759.8 | -2.598004359 | 0.00277097 |
| M358T247.pos | Oxybutynin | 1.94958428 | 358.2588 | Up | 3132767.88 | 18790585.9 | 2.584500262 | 0.01041185 |
| M332T324.neg | Glu-Gly-Glu | 1.86739662 | 332.099 | Down | 9184696.56 | 1531654.34 | -2.584141312 | 0.01023134 |
| M586T325.pos | Glucopyranosiduronic acid | 1.56845042 | 586.1877 | Down | 42364875 | 7191692.1 | -2.558465451 | 0.04149242 |
| M215T26.neg | Nepodin | 1.93078883 | 215.0854 | Down | 31368677.7 | 5649211.81 | -2.473203219 | 0.00625641 |
| M593T366.neg | Poncirin | 1.97770714 | 593.1876 | Down | 2947585.1 | 564982.004 | -2.383256645 | 0.00281396 |
| M202T317_1.pos | Simazine | 2.23571798 | 202.071 | Down | 138420169 | 27344714.9 | -2.339720239 | 0.00068884 |
| M221T300.neg | Melibiose | 1.6930473 | 221.0667 | Down | 59795527.4 | 11895608.6 | -2.329608497 | 0.02241768 |
| M523T65.neg | Asiatic acid | 1.94025918 | 523.3048 | Up | 37170223.1 | 172501361 | 2.214388489 | 0.01179494 |
| M570T471.pos | Harringtonine | 1.72811312 | 570.2145 | Down | 3529484.83 | 768007.07 | -2.200266125 | 0.02495423 |
| M274T23.neg | Ala-Trp | 1.78990873 | 274.1065 | Down | 19938900.9 | 4434009.48 | -2.168902128 | 0.01873421 |
| M248T264.pos | Lys-Thr | 2.16239374 | 248.1394 | Up | 8168670.03 | 35572389.2 | 2.122584765 | 0.00106241 |
| M455T206.pos | 2'-deoxycytidine | 2.1058036 | 455.1888 | Up | 9754891.97 | 42404473.2 | 2.120018661 | 0.00248212 |

Note: DEMs: differently expression metabolites; VIP: Variable Importance in Projection; FMT: Fecal microbiota transplantation group; MOD: Gas explosion model group; pos: positive ion mode; neg: negative ion mode.

Table S7 Top 20 DEMs in positive and negative ion modes were screened between AF and ABX groups using metabonomics.

| ID | Name | VIP | m/z | Regulation | Mean ABX | Mean AF | log2FoldChange | *p*value |
| --- | --- | --- | --- | --- | --- | --- | --- | --- |
| M271T391_2.pos | Tetraene-3,17. beta. | 2.33081994 | 271.1652 | Up | 7899774.73 | 4131527345 | 9.030647987 | 3.87E-08 |
| M419T402.pos | Hc toxin | 2.26086385 | 419.214 | Down | 93884622.4 | 462538.018 | -7.665173112 | 3.26E-05 |
| M295T395_1.pos | Cinchonidine | 2.30143384 | 295.1653 | Up | 3260356.28 | 640267563 | 7.617501486 | 8.93E-05 |
| M324T399_2.pos | Pentamidine | 2.28557523 | 324.1806 | Up | 3346586.06 | 346447989 | 6.693804857 | 6.56E-05 |
| M297T384_2.pos | 17alpha-ethynylestradiol | 2.30525734 | 297.1808 | Up | 3234706.23 | 314972104 | 6.605445556 | 2.21E-05 |
| M346T347.pos | Thyrotropin | 2.29419918 | 346.1649 | Up | 2303062 | 198773732 | 6.431430056 | 7.40E-07 |
| M209T412.pos | Pilocarpine | 2.28660994 | 209.1284 | Up | 3007267.15 | 195709268 | 6.024115233 | 1.08E-05 |
| M309T387.pos | Vincanidine | 2.28106412 | 309.1808 | Up | 2714641.05 | 133735553 | 5.622477796 | 4.97E-05 |
| M223T311.pos | Zectran | 2.31486312 | 223.1441 | Up | 9632005.78 | 453147876 | 5.556001853 | 7.48E-08 |
| M389T444.pos | Val-Asp-Arg | 2.28570359 | 389.1921 | Up | 1887490.34 | 87192356.7 | 5.529660511 | 2.75E-05 |
| M176T356_1.pos | Carisoprodol | 2.22277305 | 176.1281 | Up | 68582792.9 | 3066061948 | 5.482396379 | 3.82E-05 |
| M329T410.pos | Propentofylline | 2.26190251 | 329.171 | Up | 2952560.97 | 130568237 | 5.466693324 | 0.00021239 |
| M250T266.pos | Alprenolol | 2.2601083 | 250.1801 | Up | 1087136.78 | 40930955.3 | 5.234586975 | 1.03E-05 |
| M299T398.pos | Olomoucine | 2.31017312 | 299.1602 | Up | 7922781.25 | 296667478 | 5.226696004 | 9.63E-07 |
| M355T396.pos | Vincamine | 2.02134573 | 355.1976 | Up | 1146153.21 | 36840710.9 | 5.006429089 | 0.0038044 |
| M232T363.pos | Aminophenazone | 2.20731373 | 232.1539 | Up | 7890064.64 | 226335981 | 4.842285018 | 3.99E-05 |
| M240T307.pos | Tert-butoxycarbonyl | 2.2467434 | 240.1229 | Up | 23812832.1 | 669798478 | 4.813915981 | 8.19E-05 |
| M248T296_2.pos | Meperidine | 2.32150074 | 248.1649 | Up | 2209232.65 | 58450466.1 | 4.725597271 | 3.10E-08 |
| M208T332.pos | Terbutaline | 2.22882066 | 208.1331 | Up | 4801178.67 | 122587787 | 4.67428282 | 0.00013627 |
| M219T566.pos | Tributylphosphine oxide | 2.16945907 | 219.17 | Up | 1291131.51 | 32572623.5 | 4.656952068 | 0.0004672 |

Note: DEMs: differently expression metabolites; VIP: Variable Importance in Projection; AF: Antibiotic removal + gas explosion + fecal microbiota transplantation group; ABX: Antibiotic clearance + gas explosion model group; pos: positive ion mode; neg: negative ion mode.
